# Virulence evolution of *Toxoplasma gondii* within a multi‐host system

**DOI:** 10.1111/eva.13530

**Published:** 2023-01-23

**Authors:** Mengyue Wang, Wen Jiang

**Affiliations:** ^1^ Department of Mechanics Huazhong University of Science and Technology Wuhan China; ^2^ Hubei Key Laboratory for Engineering Structural Analysis and Safety Assessment Wuhan China

**Keywords:** adaptive dynamics, cat‐mouse interaction, evolutionary bifurcation, evolutionary ecological feedback, multi‐host system, virulence evolution

## Abstract

Current research on the virulence evolution of *Toxoplasma gondii* is mainly conducted via experiments, and studies using mathematical models are still limited. Here, we constructed a complex cycle model of *T. gondii* in a multi‐host system considering multiple transmission routes and cat‐mouse interaction. Based on this model, we studied how the virulence of *T. gondii* evolves with the factors related to transmission routes and the regulation of infection on host behavior under an adaptive dynamics framework. The study shows that all factors that enhance the role of mice favored decreased virulence of *T. gondii*, except the decay rate of oocysts that led to different evolutionary trajectories under different vertical transmission. The same was true of the environmental infection rate of cats, whose effect was different under different vertical transmission. The effect of the regulation factor on the virulence evolution of *T. gondii* was the same as that of the inherent predation rate depending on its net effect on direct and vertical transmissions. The global sensitivity analysis on the evolutionary outcome suggests that changing the vertical infection rate and decay rate was most effective in regulating the virulence of *T. gondii*. Furthermore, the presence of coinfection would favor virulent *T. gondii* and make evolutionary bifurcation easy to occur. The results reveal that the virulence evolution of *T. gondii* had a compromise between adapting to different transmission routes and maintaining the cat‐mouse interaction thereby leading to different evolutionary scenarios. This highlights the significance of evolutionary ecological feedback to evolution. In addition, the qualitative verification of *T. gondii* virulence evolution in different areas by the present framework will provide a new perspective for the study of evolution.

## 1 INTRODUCTION

The compartment model is widely used to comprehend the selective pressures acting on parasites (Frank, 1996) when studying the virulence evolution of parasites (Boldin & Kisdi, 2012; Day, 2002; Lipsitch et al., 1996; Morozov & Adamson, 2011; Morozov & Best, 2012). However, the current research on the virulence evolution of *Toxoplasma gondii* is mainly through experiments (Amouei et al., 2020; Galal et al., 2019; Galeh et al., 2021; Shwab et al., 2014, 2018) and studies from a mathematical perspective are still limited (Deng et al., 2021). *T. gondii* is regarded as one of the most successful parasites in the world due to its worldwide distribution (Dubey, 2010) and its high prevalence in the human population (Tenter et al., 2000). Such success is attributed to the fact that *T. gondii* can infect nearly all warm‐blooded mammals via multiple transmission routes, such as a contact with the environment polluted by the oocysts released by infected felids like cats (i.e., environmental transmission), the ingestion of undercooked meat harboring tissue cysts (i.e., direct transmission), and mother‐to‐child transmission (i.e., vertical transmission; Dubey, 1998, 2010; Hill & Dubey, 2002; Tenter et al., 2000). These epidemiological details have been considered in some studies on mathematical modeling of *T. gondii* to study the control of *T. gondii* transmission under different transmission scenarios, such as cat–human transmission (González‐Parra et al., 2009; Pei et al., 2018), cat–environment transmission (Cen et al., 2014; Feng et al., 2013), and cat–environment–mouse transmission (Jiang et al., 2012; Lelu et al., 2010; Turner et al., 2013). However, none of them involve the research on the virulence evolution of *T. gondii*, which is of great significance for the long‐term control and intervention of *T. gondii*. Thus, it is necessary to construct a model that explicitly considers multiple hosts with different transmission routes and interactions between hosts to study the virulence evolution of *T. gondii*.

The inclusion of multiple hosts is important for understanding the transmission dynamics of infectious diseases (Auld et al., 2017). It plays an important role in maintaining genetic diversity both in hosts and parasites (Kawecki, 1994) given that most parasites can infect more than one type of host (Taylor et al., 2001). A probable reason for this is that the high genetic variability of parasites makes it possible to be associated with different hosts and have the ability to infect them by different transmission routes (Woolhouse et al., 2001). The transmission route is a determinant of not only the spread of disease (Turner et al., 2013) but also the evolution of parasites (Regoes et al., 2000). Both the virulence‐transmission trade‐offs among hosts with distinct transmission routes (Woolhouse et al., 2001) and the effectiveness of between‐host transmission in a heterogeneous population (Betancourt et al., 2013; Gandon, 2004) can influence virulence evolution, leading to a higher (or lower) level of virulence and even evolutionary bifurcation.

In addition, biotic factor such as predation is another important factor affecting virulence evolution (Choo et al., 2003; Morozov & Adamson, 2011; Morozov & Best, 2012). As part of host mortality, the general idea that increasing host mortality favors increased virulence (Anderson & May, 1982) also holds if such an increase is induced by static predators in a classical SI model (Morozov & Adamson, 2011). However, if parasite virulence influences host mortality in ways other than parasitism, more complicated scenarios can arise (Williams & Day, 2001). For example, evolving toward decreased virulence (Choo et al., 2003) and evolutionary bifurcation (Morozov & Best, 2012) are all possible if increased parasite virulence increases the predation risk, which can be attributed to the regulation of infection on host behavior (Ajai et al., 2007; Rigby & Jokela, 2000).

Although a general model with multiple hosts for different special cases of diseases has been constructed by Gandon (Gandon, 2004) to illustrate the diversity of the host–parasite life cycle, it is invalid for *T. gondii*. The modeling of *T. gondii* should explicitly consider environmental transmission and the significant role of predation induced by cat–mouse interaction (Ajai et al., 2007; Turner et al., 2013). Thus, the present paper aims to build a complex cycle model of *T. gondii* with multiple transmission routes of *T. gondii* and interactions between different hosts. This model will be used to study how the virulence of *T. gondii* evolves with the factors related to transmission routes and the regulation of infection on host behavior under the framework of adaptive dynamics.

## 2 MATHEMATICAL MODEL

Herein, a complex cycle model will be constructed to model the transmission dynamics of *T. gondii* as realistically as possible. Such a model should explicitly contain all representative hosts: definitive hosts (e.g., cats, the only source of oocysts released into the environment [Dubey, 1998; Hill & Dubey, 2002]), intermediate hosts (e.g., mice, the prey of cats), other intermediate hosts (e.g., pigs, cattle, and sheep), and humans. However, one thing we need to emphasize here is that other intermediate hosts and humans can neither change the transmission and maintenance of *T. gondii* in the system nor affect the final evolutionary scenario due to a unidirectional feedback between infection and these two types of hosts. Therefore, our model only involves the other two types of hosts (i.e., cats and mice) that are crucial to both the transmission and evolution dynamics of *T. gondii* (Shwab et al., 2018; Turner et al., 2013).

### 2.1 The transmission dynamics of *T. gondii*

Based on the epidemiological characteristics of *T. gondii*, we made the following assumptions to construct the model as simply as possible: (1) the vertical transmission in cats can be neglected (Dubey & Hoover, 1977); (2) the infected cats have the probability to recover (Tenter et al., 2000); (3) mice are mostly likely to be infected vertically and environmentally (Hill et al., 2005; Hill & Dubey, 2002); and (4) the infection in mice can lead to a nonnegligible mortality (i.e., virulence; Dubey, 1998) and regulate mouse behavior, making them more likely to be caught by cats (Ajai et al., 2007). On the basis of these assumptions, we use the following ordinary differential equations to describe the transmission dynamics of *T. gondii* (Equation (1)).
(1)

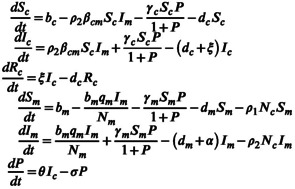

Here, two types of hosts, cats and mice, are distinguished by subscripts c and m, respectively. The total population of each host ($N_{c}$, $N_{m}$) consists of the susceptible ($S_{c}$, $S_{m}$) and the infected components ($I_{c}$, $I_{m}$), where cats contain an additional component $R_{c}$, those recovering from infection at a rate ξ. Each type of host has its own population birth rate ($b_{c}$, $b_{m}$) and per capita natural death rate ($d_{c}$, $d_{m}$), both of which are regarded as constants. The probability of the environmental and direct infection of cats by exposure to a contaminated environment and predation on infected mice is $\gamma_{c}$ and $\beta_{cm}$, respectively. For mice, the chances of being infected environmentally and vertically are $\gamma_{m}$ and $q_{m}$. The mortality of mice due to infection (i.e., virulence) is measured by α. The effect of infection on mouse behavior is represented by different predation rates of cats on susceptible and infected mice ($\rho_{1}$, $\rho_{2}$). The oocysts (P) that pollute the environment are released by infected cats at a rate θ and decay at a rate σ.

System (1) always has a disease‐free equilibrium $W_{0}=\left(S_{c}^{0},0,0,S_{m}^{0},0,0\right)$, where $S_{c}^{0}=N_{c}^{0}=b_{c}/d_{c}$ and $S_{m}^{0}=N_{m}^{0}=b_{m}/\left(d_{m}+\rho_{1}N_{c}\right)$. The endemic equilibrium of system (1) is $W_{+}=\left({\hat{S}}_{c},{\hat{I}}_{c},{\hat{R}}_{c},{\hat{S}}_{m},{\hat{I}}_{m},\hat{P}\right)$ (see Appendix A for more details), and the total numbers of cats and mice now are ${\hat{N}}_{c}={\hat{S}}_{c}+{\hat{I}}_{c}+{\hat{R}}_{c}\equiv N_{c}^{0}\equiv b_{c}/d_{c}$ and ${\hat{N}}_{m}={\hat{S}}_{m}+{\hat{I}}_{m}$. Whether the endemic equilibrium $W_{+}$ exists is determined by the basic reproduction number $R_{0}$, the spectral radius of the next‐generation matrix in the theory of van den Driessche and Watmough (van den Driessche & Watmough, 2002), which can be derived from system (1) and expressed as (see Appendix A for more details)

(2) $$\begin{array}{c} R_{0}=\frac{1}{2}\left(S_{c}^{0}E_{c}+\frac{V_{m}}{N_{m}^{0}}\right)+\frac{1}{2}\sqrt{{\left(S_{c}^{0}E_{c}-\frac{V_{m}}{N_{m}^{0}}\right)}^{2}+4S_{c}^{0}D_{c}S_{m}^{0}E_{m}}\\ E_{c}=\frac{\gamma_{c}\theta }{\sigma \left(d_{c}+\xi \right)};D_{c}=\frac{\rho_{2}\beta_{\mathrm{cm}}}{d_{m}+\alpha +\rho_{2}N_{c}^{0}};E_{m}=\frac{\gamma_{m}\theta }{\sigma \left(d_{c}+\xi \right)};V_{m}=\frac{b_{m}q_{m}}{d_{m}+\alpha +\rho_{2}N_{c}^{0}} \end{array}.$$

where $E_{c}$ and $D_{c}$ are the environmental and direct transmission intensities of cats at the disease‐free equilibrium, respectively. Similarly, $E_{m}$ and $V_{m}$ represent the environmental and vertical transmission intensities of mice. If $R_{0}>1$, the endemic equilibrium $W_{+}$ exists. Otherwise, there is only a disease‐free equilibrium $W_{0}$.

### 2.2 The evolutionary dynamics of *T. gondii*

The processes of within‐host replication of pathogens and transmission routes are interrelated (Auld et al., 2017; Guidot et al., 2014; Lievens et al., 2018). Both processes can evolve through mutation and selection, but such changes are often correlated. Increasing pathogen replication will increase transmissibility but also increase the risk of host death given that pathogen replication will consume host resources and have adverse effects on them (Anderson & May, 1982). Such correlations can be represented by tradeoff functions. Here, we took toxicity τ, the adverse effect from *T. gondii*, as the evolutionary trait, and constructed the relations between τ and the transmission‐related parameters and host mortality due to infection. That is, the infection rates $\beta_{cm}$, $q_{m}$, $\gamma_{c}$, $\gamma_{m}$, release rate θ, and virulence α will increase with increasing τ. In addition, we made the decay rate of oocysts σ decrease with the increase of τ based on the classical “Curse of the Pharoah” hypothesis (Bonhoeffer et al., 1996; i.e., the more toxic the pathogen is, the longer it will survive in the environment). Given that these parameters cannot vary indefinitely with τ, we took the forms of saturation functions (Boldin & Kisdi, 2012; Day, 2002), that is,

(3) $$\begin{array}{c} \alpha \left(\tau \right)=1-e^{-c_{\alpha }\tau };\;\beta_{\mathrm{cm}}\left(\tau \right)=\frac{\tau }{\tau +\kappa_{\beta }};\;q_{m}\left(\tau \right)=\frac{\tau }{\tau +\kappa_{q}};\\ \gamma_{c}\left(\tau \right)=\frac{\tau }{\tau +\kappa_{\gamma_{c}}};\;\gamma_{m}\left(\tau \right)=\frac{\tau }{\tau +\kappa_{\gamma_{m}}};\;\theta \left(\tau \right)=\frac{\tau }{\tau +\kappa_{\theta }};\;\sigma \left(\tau \right)=e^{-\mathrm{u\tau}}+v; \end{array}$$

For simplicity, the recovery rate ξ and predation rate $\rho_{1}$, $\rho_{2}$ are thought to be constant.

Therefore, the evolution of *T. gondii* is depicted by the mutation in toxicity. Based on the idea that there is a separation of transmission and evolutionary dynamics on time scales in the adaptive dynamics framework (Geritz et al., 1998), we suppose that when the resident *T. gondii* with toxicity $\tau_{r}$ is at the endemic equilibrium $W_{+}$, a few mutant *T. gondii* with toxicity $\tau_{m}$ that slightly differs in $\tau_{r}$ will appear in the system and compete with the resident. The competition between them can be regarded as an invasion of the mutant to the resident system that has reached its endemic equilibrium. The invasion dynamics of the mutant *T. gondii* can therefore be described as follows:
(4)

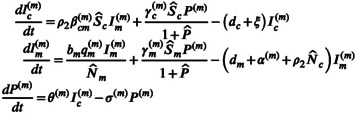

Here, the superscript “(m)” is associated with the mutant *T. gondii*. The transmission‐related parameters and virulence are functions of $\tau_{m}$ satisfying those relations in Equation (3). It should be noted that the number of the mutant individuals is rare compared with the resident population (i.e., $I_{c}^{\left(m\right)}\ll {\hat{N}}_{c}$, $I_{m}^{\left(m\right)}\ll {\hat{N}}_{m}$, $P^{\left(m\right)}\ll \hat{P}$), thus, its effect on the number of the population in the current state can be neglected (Dercole & Rinaldi, 2008).

Whether the mutant can successfully invade the resident system is determined by the invasion fitness of the mutant *T. gondii*, that is, the invasion reproduction number $R^{m}$, which can be derived by the next‐generation matrix method (van den Driessche & Watmough, 2002) on the basis of Equation (4) (see Appendix B for more details):

(5) $$R^{m}\left(\tau_{r},\tau_{m}\right)=\frac{1}{2}\left(\frac{{\hat{S}}_{c}E_{c}^{\left(m\right)}}{1+\hat{P}}+\frac{V_{m}^{\left(m\right)}}{{\hat{N}}_{m}}\right)+\frac{1}{2}\sqrt{{\left(\frac{{\hat{S}}_{c}E_{c}^{\left(m\right)}}{1+\hat{P}}-\frac{V_{m}^{\left(m\right)}}{{\hat{N}}_{m}}\right)}^{2}+\frac{4{\hat{S}}_{c}D_{c}^{\left(m\right)}{\hat{S}}_{m}E_{m}^{\left(m\right)}}{1+\hat{P}}}$$

If $R^{m}>1$, the mutant can invade the resident system and replace the resident. Otherwise, it cannot invade the system. That is, *T. gondii* with higher invasion fitness will succeed (Geritz et al., 1998), so the direction of the evolution of *T. gondii* is to maximize its invasion reproduction number. This implies that the evolutionary singularity $\tau^{*}$ satisfying

(6) $${\left.\frac{\partial R^{m}\left(\tau_{r},\tau_{m}\right)}{\partial \tau_{m}}\right|}_{\tau_{r}=\tau_{m}=\tau *}=0$$

is a candidate for the evolutionary outcome that can eventually be reached asymptotically after continuous mutation. Whether $\tau^{*}$ refers to this achievable evolutionary outcome needs further judgment. For this reason, the evolutionary singularity is classified into three types according to Geritz et al. (1998):

1. When the singularity can be achieved and cannot be invaded by any mutants, that is, A+B<0 and A<0, it is a continuous stable strategy (CSS);
2. When the singularity can be achieved and can be invaded by mutants, that is, A+B<0 and A>0, it is a branch point;
3. When the singularity cannot be achieved, that is, A+B>0, it is a repeller,

where

(7) $$A={\left.\frac{\partial^{2}R^{m}\left(\tau_{r},\tau_{m}\right)}{\partial \tau_{m}^{2}}\right|}_{\tau_{m}=\tau_{r}=\tau^{*}};\;{\left.\;B=\frac{\partial^{2}R^{m}\left(\tau_{r},\tau_{m}\right)}{\partial \tau_{r}\partial \tau_{m}}\right|}_{\tau_{m}=\tau_{r}=\tau^{*}}.$$

Obviously, the evolutionary outcome is highly dependent on the factors related to transmission routes and the regulation of infection on host behavior, whose effects on the virulence evolution of *T. gondii* will be discussed in detail in the next section.

### 2.3 The determination of the values and ranges of parameters

Parameters in the present model are obtained from the data of epidemiological survey and infection experiments of *T. gondii* and then rationalized based on the existing framework (the second column of Table 1). Considering the limitation of collected data, the ranges of these parameters are further expanded to make up for this limitation (the fourth column of Table 1).

**TABLE 1:** Values of the parameters used in Equation (1) and source.

Parameter	Value (per week)	Source	Value used in the model (per week)
$b_{c}$	^a^		2.25
$d_{c}$	[0.0096, 0.019]	Courchamp et al. (1995), Gotteland et al. (2014), Jiang et al. (2012), Lelu et al. (2010)	0.015
$b_{m}$	^a^		337.5
$d_{m}$	[0.038, 0.048]	Gotteland et al. (2014), Jiang et al. (2012), Lelu et al. (2010)	0.045
ξ	^b^		0.2
$\rho_{1}$	[2.5, 64] × 10^−5^	Baker et al. (2003, 2005), Barratt (1998), Kays and DeWan (2004)	[1, 80] × 10^−5^
α	[0.040, 1]	Dubey (1996, 2006), Dubey et al. (1997), Dubey and Frenkel (1973)	[0.001, 1]
$\beta_{cm}$	[0.56, 0.99]	Dubey (2001, 2006), Dubey and Frenkel (1972, 1976)	[0.5, 1]
$q_{m}$	[0.33, 0.91]	Dubey and Frenkel (1998), Hide (2016)	[0.3, 1]
$\gamma_{c}$	^c^		[0.1, 1]
$\gamma_{m}$	[0.30, 0.96]	Dubey (1996, 2006), Dubey et al. (1997), Dubey and Frenkel (1973)	[0.1, 1]
θ	[0.061, 0.40]	Lelu et al. (2010)	[0.03, 0.65]
σ	[0.019, 0.70]	Dubey et al. (1998), Gotteland et al. (2014), Jiang et al. (2012), Lelu et al. (2010)	[0.015, 1]

^a^ The birth rate is determined by $b_{c}=N_{c}d_{c}$ ($b_{m}=N_{m}d_{m}$) in the absence of infection and predation.

^b^ Is an estimated value.

^c^ We suppose that $\gamma_{c}$ can also vary within the same range as $\gamma_{m}$.

#### 2.3.1 The death rates and population birth rates of cats and mice

There are approximately 150–1100 cats (Baker et al., 2003, 2005; Barratt, 1998; Courchamp et al., 1995) and 2000–8100 mice (Baker et al., 2003, 2005) per km^2^ with survival times of 1–2 years (Courchamp et al., 1995; Gotteland et al., 2014; Jiang et al., 2012; Lelu et al., 2010) and 0.4–0.5 years (Gotteland et al., 2014; Jiang et al., 2012; Lelu et al., 2010), respectively. Thus, the death rate per week of cats is [0.0096, 0.019] and that of mice is [0.038, 0.048]. Here, we adopt $d_{c}$ = 0.015 and $d_{m}$ = 0.045 as the death rates of cats and mice per week and take $N_{c}$ = 150 and $N_{m}$ = 7500 as the total numbers of each host in the absence of infection and predation. In this case, the population birth rates of cats and mice are 2.25 and 337.5 per week, which can be obtained by $b_{c}=N_{c}d_{c}$ and $b_{m}=N_{m}d_{m}$.

#### 2.3.2 Predation rate

Approximately 10.2–66.48 mice are caught by a cat per year (Baker et al., 2005; Barratt, 1998; Kays & DeWan, 2004), which is equivalent to 0.2–1.28 mice being caught per week (i.e., 10.2/52–66.48/52). Combining with the information that the number of mice is 2000–8100 (Baker et al., 2003, 2005), the probability of a mouse being caught by a cat per week varies in [2.5, 64] × 10^−5^ (i.e., 0.2/8100–1.28/2000). We take [1, 80] × 10^−5^ as the variation range of the inherent predation rate $\rho_{1}$ in this model.

#### 2.3.3 Virulence and the transmission‐related parameters

Here, the same method as Turner et al. (2013) is used to obtain virulence and the transmission‐related parameters. In *T. gondii* infection experiments, the dead mice induced by infection accounts for 10%–100% of the infected mice during 8–18 days (Dubey, 1996, 2006; Dubey et al., 1997; Dubey & Frenkel, 1973). Therefore, the virulence of *T. gondii* to mice varies from 1–(1–0.1)^7/18^ = 0.040 to 1–(1–1)^7/8^ = 1 per week and we choose the range of the virulence α to be [0.001, 1] herein. The proportion of cats infected by the tissue cysts of infected mice varies from 69% to 97% (Dubey, 2006; Dubey & Frenkel, 1972, 1976) during 5–10 days (Dubey, 2001, 2006; Dubey & Frenkel, 1972, 1976). Therefore, the probability of cats infected by tissue cysts per week ranges in [0.56, 0.99], and we adopt [0.5, 1] for the range of the direct infection rate $\beta_{cm}$. Similarly, approximately 60%–98% mice are infected by oocysts during 8–18 days (Dubey, 1996, 2006; Dubey et al., 1997; Dubey & Frenkel, 1973). Therefore, the environmental infection rate of mice $\gamma_{m}$ per week changes in [0.30, 0.96]. We take [0.1, 1] as the range of $\gamma_{m}$ and suppose that the environmental infection rate of cats $\gamma_{c}$ also varies in the same range. According to the vertical infection experiments of mice, the weekly infection rate varies in [0.33, 0.91] (Dubey & Frenkel, 1998; Hide, 2016). We adopt [0.3, 1] for the range of the vertical infection rate $q_{m}$. The probability of oocysts being released by infected cats is [0.0089, 0.07] per day (Lelu et al., 2010). We calculate that it is equivalent to [0.061, 0.40] per week and take [0.03, 0.65] as the range of the release rate of oocysts θ. Given that the oocysts can survive for dozens of days to several months outdoors (Dubey et al., 1998; Gotteland et al., 2014; Jiang et al., 2012; Lelu et al., 2010), we assume that its survival time is 10–365 days. Thus, the decay rate of oocysts σ changes in [0.019, 0.70] per week and we choose the range of it to be [0.015, 1].

We take the following way to assign the value or value ranges of the coefficients in Equation (3) to meet the value ranges of those parameters in Table 1. The toxicity *τ* is thought to vary from 0.05 to 10 and ν is set to be the lower limit of the decay rate (i.e., ν = 0.015). We equally divide the value ranges of α, $\beta_{cm}$, $q_{m}$, $\gamma_{m}$, $\gamma_{c}$, θ, σ, and τ into 2000 nonoverlapping intervals and carry out random sampling in each interval. Then, we sort these samples according to the monotonic relations between these parameters and τ described by Equation (3) and obtain the maximums and minimums of the virulence‐related and transmission‐related coefficients. Finally, we repeat such sampling procedures 100 times to obtain the average values of those maximums and minimums as their value ranges, that is,

(8) $$\begin{array}{c} c_{\alpha }\in \left[0.024,0.46\right];\kappa_{\beta }\in \left[0.051,1.2\right];\kappa_{q}\in \left[0.11,2.9\right];\\ \kappa_{\gamma_{m}}=\kappa_{\gamma_{c}}\in \left[0.47,7.1\right];\kappa_{\theta }\in \left[1.8,10.2\right];u\in \left[0.12,0.85\right] \end{array}.$$

In this paper, we take $c_{\alpha }$ = 0.08 to ensure that virulence varies moderately with toxicity and further expand the ranges of $\kappa_{\beta }$, $\kappa_{q}$, $\kappa_{\gamma_{m}}$, $\kappa_{\gamma_{c}}$, $\kappa_{\theta }$, and u to that shown in Table 2 to study the effects of different factors on the virulence evolution of *T. gondii* on a large scale.

**TABLE 2:** Value or value ranges of the coefficients in Equation (3).

Coefficients	Value or value range	Coefficients	Value or value range
$\kappa_{\beta }$	[0, 1.5]	$\kappa_{\theta }$	[0, 12]
$\kappa_{q}$	[0, 4]	u	[0.1, 3]
$\kappa_{\gamma_{c}}\left(\kappa_{\gamma_{m}}\right)$	[0, 10]	$c_{\alpha }$	0.08

## 3 RESULTS

In this section, we will first study how the virulence of *T. gondii* evolves in response to transmission routes and the regulation of infection on host behavior. The former are closely related to its infection rates, which can be represented by the infection rate‐related parameters $\kappa_{q}$, $\kappa_{\beta }$, $\kappa_{\gamma_{c}}$, and $\kappa_{\gamma_{m}}$. In addition, the transmission route characterized by its transmission intensity is also associated with other transmission‐related parameters, such as the predation rate and the release and decay rates of oocysts, which can be reflected by $\rho_{2}$, $\kappa_{\theta }$, and u, respectively. The latter is shown as different predation rates on susceptible and infected mice ($\rho_{1}$ and $\rho_{2}$), according to Section 2.1. Here, we treat such a difference as a constant Δρ that is defined as the regulation factor and satisfies $\rho_{2}$ = $\rho_{1}$ + Δρ, and make $\rho_{2}$ = $\rho_{1}$ when regarding it as a transmission‐related factor, but distinguish $\rho_{1}$ and $\rho_{2}$ by Δρ when speaking of the regulation of infection on host behavior. Then, we will further give a global sensitivity analysis on the evolutionary outcome of *T. gondii* when these factors vary globally and simultaneously. Finally, given that it is possible for hosts to be co‐infected by multiple *T. gondii* strains, we will also consider the situation of coinfection by both resident and mutant strains during evolution.

### 3.1 The influences of different transmission routes on the virulence evolution of *T. gondii*

#### 3.1.1 The roles of vertical and direct transmissions

**FIGURE 1:**
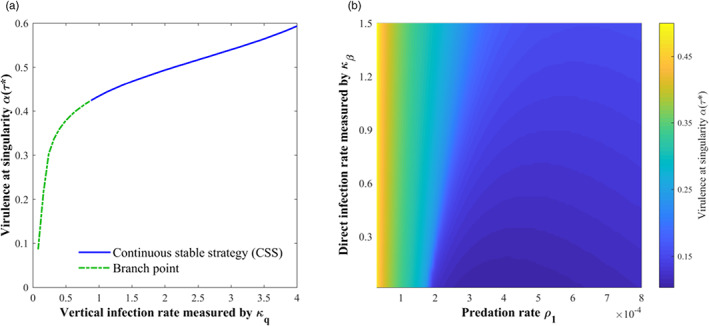
The effects of the factors related to vertical and direct transmissions on the virulence evolution of *Toxoplasma gondii*: Variation of virulence with (a) vertical infection rate‐related parameter $\kappa_{q}$, (b) direct infection rate‐related parameter $\kappa_{\beta }$ and predation rate $\rho_{1}$. Parameters in (a) $c_{\alpha }$ = 0.08, ξ = 0.2, u = 1, ν = 0.015, $\kappa_{\beta }$ = 0.5, $\rho_{2}$ = $\rho_{1}$ = 6 × 10^−4^, $\kappa_{\theta }$ = 1, $\kappa_{\gamma_{c}}$= 1, $\kappa_{\gamma_{m}}$= 1; (b) $\kappa_{q}$ = 0.1, $\rho_{2}$ = $\rho_{1}$ and others are the same as those in (a).

Figure 1 examines how the virulence of *T. gondii* evolves with vertical and direct transmissions, whose transmission intensities are related to both its own infection rate and the predation rate (see Equation (B.5)). When the vertical infection rate gradually increased ($\kappa_{q}$↓), a stable highly virulent strain (i.e., CSS) dominant in the evolutionary scenario would gradually decrease its virulence and transform to the scenario with the coexistence of multiple strains (i.e., branch point; Figure 1a). The increase in the vertical infection rate can not only enhance the vertical transmission intensity but also directly increase the number of infected mice. This will enhance the role of infected mice as the source of infection transmitted vertically in the complex cycle. Therefore, evolution will select the *T. gondii* with low virulence to ensure that mice can transmit the infection vertically to their offspring before they die. The further increase in the vertical infection rate will lead to a balance between distinct transmission routes and result in evolutionary bifurcation making multiple strains coexist.

When the direct infection rate increased ($\kappa_{\beta }$↓), there was a decrease in the virulence of *T. gondii* (Figure 1b). The direct transmission caused by the predation of cats on infected mice also highlights the role of mice as the source of infection in the cat‐mouse interaction. Thus, the enhancement of direct transmission through increasing the direct infection rate will favor decreased virulence of *T. gondii* ensuring that infected mice can be preyed on by cats before death.

However, different from the vertical and direct infection rates, the effect of the predation rate, $\rho_{1}$, on the virulence evolution of *T. gondii* was not monotonic (Figure 1b). Increasing the predation rate acts on both vertical and direct transmissions. This leads to a weakening of vertical transmission but an enhancement of direct transmission. The net effect of increasing the predation rate therefore depends on which effect is larger. When $\rho_{1}$ is small, its effect on reducing vertical transmission can be neglected and its effect on increasing direct transmission is dominant, thereby selecting for low virulent *T. gondii*. With a further increase of the predation rate, it will have a greater effect on vertical transmission than direct transmission, which will make *T. gondii* evolve in the direction of increased virulence.

#### 3.1.2 The role of environmental transmission

**FIGURE 2:**
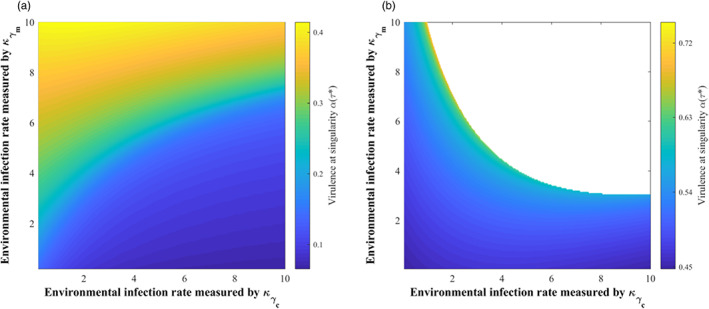
The effects of the environmental infection rate‐related parameters $\kappa_{\gamma_{c}}$ and $\kappa_{\gamma_{m}}$ on the virulence evolution of *Toxoplasma gondii* under different vertical infection rates: (a) $\kappa_{q}$ = 0.1, $\kappa_{\beta }$ = 1, $\rho_{2}$ = $\rho_{1}$ = 3.5 × 10^−4^, $\kappa_{\theta }$ = 1, and others are the same as those in Figure 1a; (b) $\kappa_{q}$ = 1 and others are the same as those in (a). The transparent area indicates that evolutionary singularity cannot be obtained.

Environmental transmission occurs in both cats and mice and its intensity of each host is related to not only its infection rate but also the release and decay rates of oocysts (see Equation (B.5)). The effects of the environmental infection rates of cats and mice on the virulence evolution of *T. gondii* under different vertical infection rates are first studied here (Figure 2). If the vertical infection rate was high ($\kappa_{q}$ = 0.1), the virulence of *T. gondii* increased monotonically with the increase of the environmental infection rate of cats ($\kappa_{\gamma_{c}}$↓; Figure 2a). In contrast, if the vertical infection rate was low ($\kappa_{q}$ = 1), the virulence of *T. gondii*, in general, would increase and then decrease (Figure 2b). However, this was not the case when the environmental infection rate of mice increased ($\kappa_{\gamma_{m}}$↓), where *T. gondii* would evolve toward decreased virulence regardless of vertical infection rate. The evolutionary outcomes brought by these two environmental infection rates were quite different. This requires explicit consideration of the different decision‐making roles of cats and mice caused by different environmental infection rates.

Cats, as the definitive hosts, are both the donors and recipients of oocysts. The increase in infected cats caused by increasing its environmental infection rate emphasizes the role of cats and the role of oocysts as the source of infection transmitted environmentally. Evolution is more likely to select for virulent *T. gondii* that facilitates the long‐term survival of oocysts. However, the potential impact of the virulence of *T. gondii* on maintaining the cat‐mouse interaction also needs further consideration due to the existence of evolutionary ecological feedback (Govaert et al., 2019; Lion, 2018; Lion & Metz, 2018). Although such an increase in virulence can reduce the lifespan of infected mice, this loss in infected mice can be compensated by the gain brought by a high vertical infection rate. Thus, the complex cycle can still be maintained. In contrast, if the vertical infection rate is low, a further increase in virulence makes mice die rapidly before being infected vertically. This will lead to a fading of cat‐mouse interaction making evolution favor low virulent *T. gondii* to maintain the number of infected mice and this complex cycle. Unlike cats, mice, as the intermediate hosts, are just the recipients of oocysts. Such an increase in infected mice caused by increasing its environmental infection rate actually further enhances the relative role of vertical infection in mice (Cressler et al., 2016; Lipsitch et al., 1996), thereby selecting for low virulent *T. gondii*.

**FIGURE 3:**
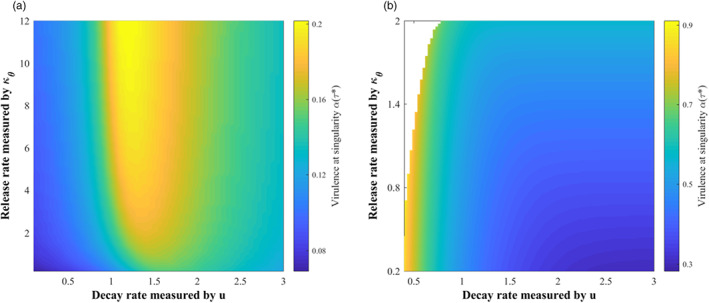
The effects of the release and decay rates of oocysts‐related parameters $\kappa_{\theta }$ and u on the virulence evolution of *Toxoplasma gondii* under different vertical infection rates: (a) $\kappa_{q}$ = 0.1, $\kappa_{\gamma_{c}}$ = 3, $\kappa_{\gamma_{m}}$ = 3; and others are the same as those in Figure 2a; (b) $\kappa_{q}$ = 1 and others are the same as those in (a). The transparent area indicates that evolutionary singularity cannot be obtained.

Then, the effects of the release and decay rates of oocysts are discussed (Figure 3). The virulence of *T. gondii* decreased with increasing the release rate ($\kappa_{\theta }$↓). It also decreased with decreasing the decay rate (u↑) when the vertical infection rate was low ($\kappa_{q}$ = 1; Figure 3b) but first increased and then decreased when the vertical infection rate was high ($\kappa_{q}$ = 0.1; Figure 3a). Increasing the release rate of oocysts can enhance the environmental transmission intensities of both cats and mice and thus the roles of cats and mice, whose effects on the virulence of *T. gondii* are different. However, the significant role of mice in all transmission routes except the environment transmission of cats makes its role outcompete that of cats and become dominant, thereby making *T. gondii* evolve toward low virulence. The same is true for decreasing the decay rate of oocysts, with the only but most important difference being that the decay rate of oocysts released by cats plays a decisive role in maintaining *T. gondii* infection. This should be considered explicitly when vertical infection is high, where the dominance of mice cannot be achieved due to the disappearance of cat‐mouse interaction caused by a rapid reduction in the survival time of oocysts (i.e., small u). It will make increased virulence of *T. gondii* be initially selected to ensure that oocysts survive long enough until the environmental infection occurs.

### 3.2 The influence of the regulation of infection on host behavior on the virulence evolution of *T. gondii*

**FIGURE 4:**
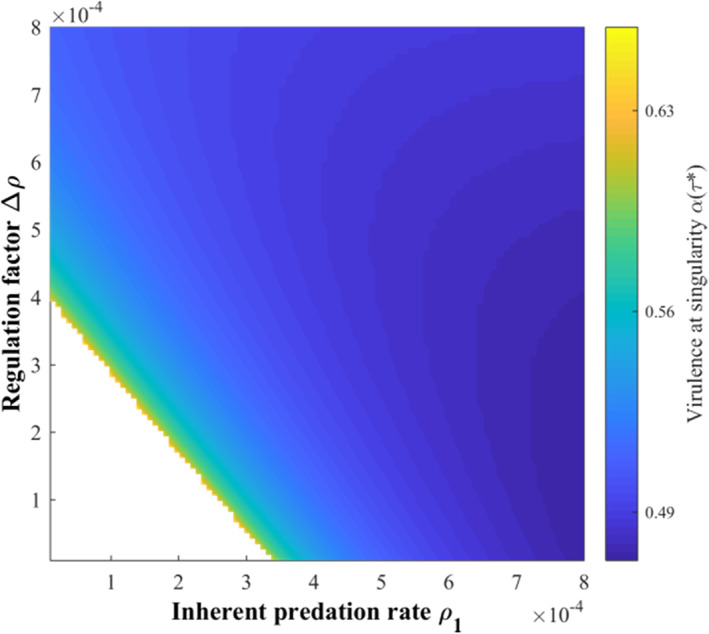
The effects of the regulation factor Δρ and the inherent predation rate $\rho_{1}$ on the virulence evolution of *Toxoplasma gondii*, where $\kappa_{q}$ = 1, $\kappa_{\beta }$ = 1, $\rho_{2}$ = $\rho_{1}$ + Δρ, $\kappa_{\theta }$ = 2, $\kappa_{\gamma_{c}}$ = 3, $\kappa_{\gamma_{m}}$ = 3, and others are the same as those in Figure 1a. The transparent area indicates that evolutionary singularity cannot be obtained.

Here, the regulation factor Δρ is introduced to depict the change in the predation rate caused by infection. The larger Δρ is, the greater the difference in the predation rate of cats on susceptible and infected mice. With the increase of the regulation factor (Δρ↑), the virulence of *T. gondii* decreased first and then increased (Figure 4). In this situation, the effect of the inherent predation rate ($\rho_{1}$) was similar to regarding it as a transmission‐related factor discussed in Subsection 3.1.1. The predation rate on infected mice $\rho_{2}$ consists of the regulation factor and the inherent predation rate. Both of them can be regarded as the factors related to direct and vertical transmissions, leading the same conclusion as that in Subsection 3.1.1 to be obtained when the predation rate is distinguished.

It should be noted that evolutionary bifurcation can occur in all cases discussed above, because the evolutionary diversity caused by mutual balance between different transmission routes (Boldin & Kisdi, 2012; Gandon, 2004) easily occurs in this system through cat‐mouse interaction. Here, the contours where each point represents either a starting point of evolutionary bifurcation or a stable evolutionary result aim to discuss the evolutionary trend of *T. gondii* virulence with different factors on a large scale.

### 3.3 Global sensitivity analysis on the evolutionary outcome of *T. gondii*

The research in Sections 3.1 and 3.2 intuitively reflects the single effect on the evolutionary outcome of each factor related to either transmission routes or the regulation of infection on host behavior. Compared with this single effect, it is more common in reality that the evolutionary outcome is subject to multiple factors simultaneously. Therefore, it is necessary to determine the sensitivity to changes in multiple factors and identify the key factors (Koutou et al., 2021; Savadogo et al., 2022; Traore et al., 2019). This is of great significance to find out the most effective prevention and control measures of *T. gondii* infection from various potential factors. Thus, a global sensitivity analysis is further performed to consider the overall impact when these factors vary simultaneously.

Here, we used Latin hypercube sampling to obtain a global sample of these factors. The eight related parameters discussed above are randomly sampled 10,000 times in the ranges presented in Table 2 and then randomly paired to form a global sample consisting of 1000 eight‐dimensional vectors, $X={\left(x_{\mathrm{ik}}\right)}_{n\times 8}={\left(x_{1},x_{2},\ldots ,x_{n}\right)}^{T},n=1000$. Therefore, the evolutionary outcome of *T. gondii* under this sample, $Y={\left(y_{\mathrm{il}}\right)}_{n\times 2}={\left(y_{1},y_{2},\ldots ,y_{n}\right)}^{T}$, can be obtained, where l = 1, 2 represent the size of virulence at singularity and the type of evolutionary singularity, respectively. The correlation coefficient between each parameter $X_{k}$ ($k\in \left\{1,\ldots 8\right\}$) and the evolutionary outcome $Y_{l}$ (l = 1, 2) is

(9) $$\text{Corr}\left(X_{k},Y_{l}\right)=\frac{\sum_{i=1}^{n}\left(x_{\mathrm{ik}}-{\bar{x}}_{k}\right)\left(y_{\mathrm{il}}-{\bar{y}}_{l}\right)}{\sqrt{\sum_{i=1}^{n}{\left(x_{\mathrm{ik}}-{\bar{x}}_{k}\right)}^{2}\sum_{i=1}^{n}{\left(y_{\mathrm{il}}-{\bar{y}}_{l}\right)}^{2}}}$$

where

(9a) $${\bar{x}}_{k}=\frac{\sum_{i=1}^{n}x_{\mathrm{ik}}}{n};{\bar{y}}_{l}=\frac{\sum_{i=1}^{n}y_{\mathrm{il}}}{n}$$

If $\text{Corr}\left(X_{k},Y_{l}\right)$ is close to 1 (or −1), there is a strong positive (or negative) linear correlation between $X_{k}$ and $Y_{l}$. Therefore, $\text{Corr}\left(X_{k},Y_{l}\right)$ is calculated to reflect the sensitivity of the evolutionary outcome to each factor.

**FIGURE 5:**
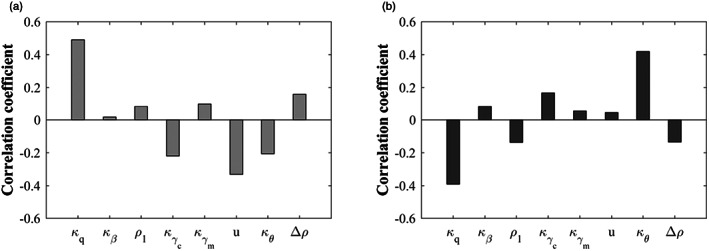
Global sensitivity analysis on the evolutionary outcome of *Toxoplasma gondii* reflected by the correlation coefficient between each parameter and (a) the size of virulence at singularity, and (b) evolutionary diversity (i.e., the type of evolutionary singularity). In (b), the branch point and CSS are characterized by 1 and 0, respectively, corresponding to the presence and absence of evolutionary bifurcation.

In Figure 5a, the overall impact on the virulence of *T. gondii* induced by each factor was basically the same as considering its single effect, except the release rate of oocysts whose impact here was opposite to its single effect. It suggests that when the interaction of multiple factors is considered, the increase in the release rate of oocysts ($\kappa_{\theta }$↓) is more likely to reinforce the important role of cats as the sole donors of oocysts thereby selecting for high virulence. Among the eight factors, the virulence of *T. gondii* was most sensitive to the vertical infection rate (i.e., $\kappa_{q}$), followed by the decay rate of oocysts (i.e., u). It indicates that the most effective way to regulate the virulence of *T. gondii* is to change the vertical infection rate of mice and the decay rate of oocysts. Figure 5b shows that decreasing the release rate of oocysts ($\kappa_{\theta }$↑) and increasing the vertical infection rate ($\kappa_{q}$↓) were more prone to evolutionary bifurcation. The change in these two factors enhances the interaction between cats and mice through decreasing the role of cats and increasing that of mice, respectively. This again reveals that changing the release rate of oocysts has a more significant effect on cats than mice when various factors vary simultaneously.

### 3.4 The situation of considering coinfection of *T. gondii*

In this section, the situation of coinfection by resident and mutant *T. gondii* during evolution is considered. This can be characterize by $I_{c}^{\left(\mathrm{rm}\right)}$ and $I_{m}^{\left(\mathrm{rm}\right)}$ in the invasion dynamics model (Equation (10)) to represent the infected cats and mice co‐infected by resident and mutant strains:
(10)

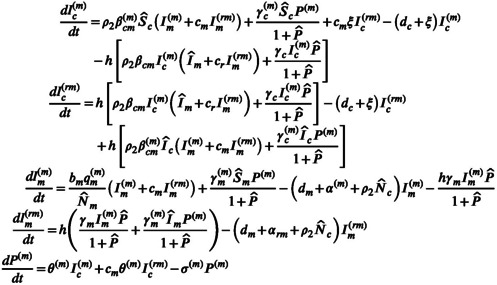




Here, the coinfection rate is represented by h. If h = 0, there is no coinfection and system (10) is equivalent to system (4). The co‐infected hosts are thought to exhibit the characteristics of the hosts infected with either resident or mutant strain (Mosquera & Adler, 1998), which is measured by $c_{r}$ and $c_{m}$, respectively. The mortality of co‐infected mice due to infection is defined as $\alpha_{rm}$. We suppose that these coinfection‐related parameters are associated with the characteristics of both resident and mutant strains (Kamiya et al., 2018; Mosquera & Adler, 1998), which are treated as functions of $\tau_{r}$ and $\tau_{m}$ in our model and satisfy the following forms:

(11) $$h=1-e^{-\kappa_{h}{\left(\tau_{r}-\tau_{m}\right)}^{2}};\;c_{r}=\frac{\tau_{r}}{\tau_{r}+\tau_{m}};\;c_{m}=\frac{\tau_{m}}{\tau_{r}+\tau_{m}};\;\alpha_{\mathrm{rm}}=1-e^{-c_{\alpha }\left(\tau_{r}+\tau_{m}\right)}.$$

The invasion reproduction number of system (10) can only be solved numerically because it is difficult to derive its analytical expression explicitly (see Appendix C for more details). Combining with Equation (6) and (7), the evolutionary outcome of *T. gondii* in the case of coinfection can be obtained.

**FIGURE 6:**
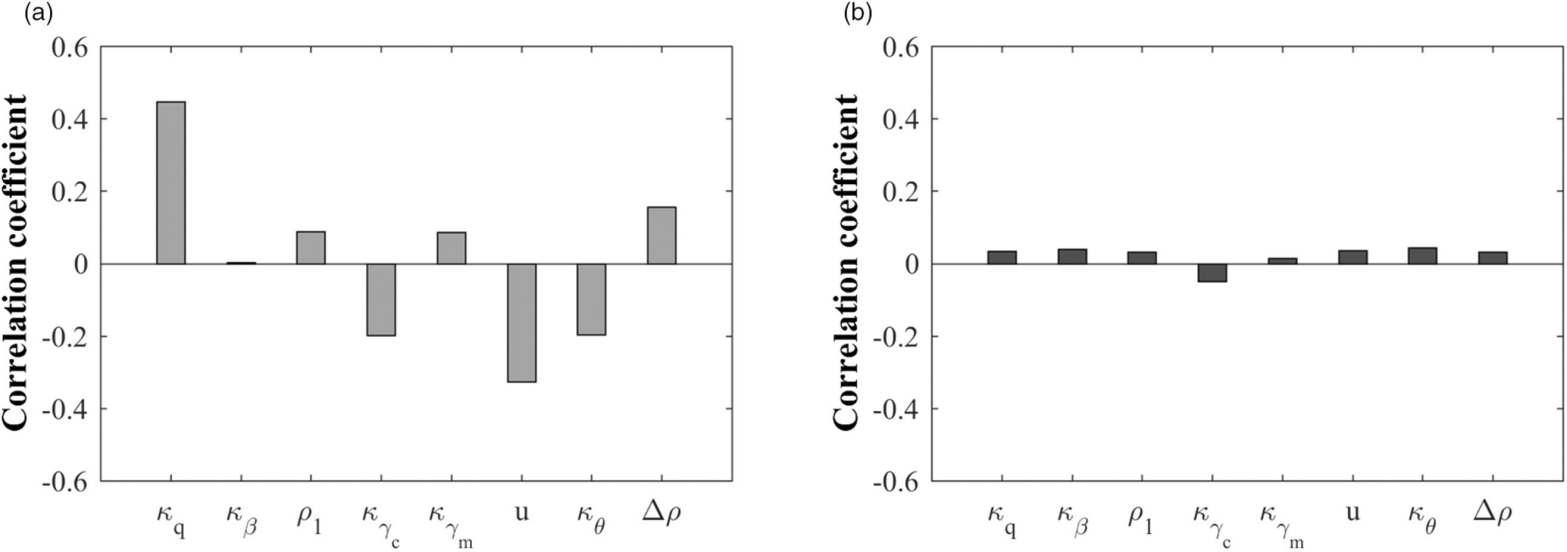
Global sensitivity analysis on the evolutionary outcome of *Toxoplasma gondii* considering coinfection. The correlation coefficient between each parameter and the size of virulence at singularity and the type of evolutionary singularity is represented by (a) and (b), respectively.

**FIGURE 7:**
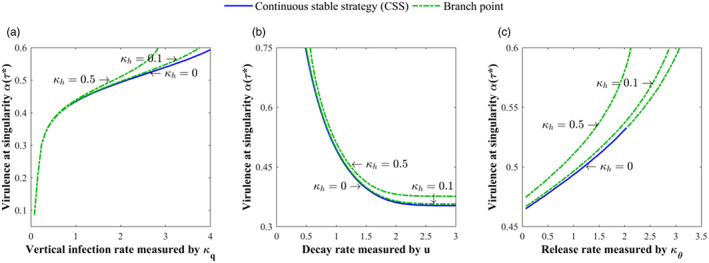
Evolutionary trend of the virulence of *Toxoplasma gondii* with (a) vertical infection rate, (b) the decay rate of oocysts, and (c) the release rate of oocysts under different coinfection rates where $\kappa_{h}$ is set to be 0, 0.1, 0.5, respectively. Parameters are the same as those in Figure 1a and $\kappa_{q}$ = 2 in (b) and (c).

Figure 6a suggests that the same conclusion on the global sensitivity analysis on the size of virulence as Figure 5a could still be obtained. In contrast, the impact of global variation in each factor on the type of evolutionary singularity was not obvious (Figure 6b), which can be intuitively reflected in Figure 7.

In Figure 7, the three factors that have significant impact on the evolutionary outcome of *T. gondii* in Section 3.3 are selected to clarify how the virulence of *T. gondii* evolves with coinfection rate. It shows that virulent *T. gondii* was more likely to be selected under high coinfection rate ($\kappa_{h}$↑) and the presence of coinfection made evolutionary bifurcation easy to occur thereby reducing the sensitivity of evolutionary diversity to various parameters. It can be revealed from Figures 6 and 7 that although the presence of coinfection increases the chance of strain coexistence making evolutionary diversity easy to occur, it does not qualitatively influence the evolutionary trend of *T. gondii* virulence with various factors.

## 4 DISCUSSION AND CONCLUSIONS

Our study has shown that in the complex cycle model of *T. gondii* with cat‐mouse interaction and multiple transmission routes, the dominance of different transmission routes could lead to different evolutionary outcomes of *T. gondii*. The present results supported the consensus that vertical and direct transmissions select for low virulent strains (Boldin & Kisdi, 2012; Day, 2002; Lipsitch et al., 1996). However, our study on environmental transmission presented a complicated picture and was not in accord with the idea that environmental transmission favors increased virulence (Boldin & Kisdi, 2012; Brown et al., 2012; Caraco & Wang, 2008; Day, 2002). This indicates that the evolutionary outcome was highly dependent on the host type and the interaction appearing in the system, which emphasizes the necessity of considering multiple transmission routes of *T. gondii* and interactions between different hosts (Dubey, 1998; Gandon, 2004; Hill & Dubey, 2002; Turner et al., 2013).

The conclusion that cats and mice are indispensable for the spread of toxoplasmosis (Dubey, 1998; Turner et al., 2013) can be demonstrated by Equation (2). This was a result of a combination of multiple transmission routes that explicitly involves cat‐mouse interaction through predation. As the sources of infection required by different transmission routes, mice and oocysts released by cats affect the virulence evolution of *T. gondii* in different directions. When mice, the source of vertical and direct transmissions, are dominant, evolution is more likely to select *T. gondii* with low virulence to ensure that mice can transmit the infection to their offspring and be preyed on by cats before they die. However, when oocysts, the source of environmental transmission, are dominant, evolution is more likely to favor highly virulent *T. gondii* that facilitates the long‐term survival of oocysts, according to the classical “Curse of the Pharoah” hypothesis (Bonhoeffer et al., 1996). *T. gondii* strains that exploit these two sources of infection to transmit face selective pressures due to different adaptations required for multiple transmission routes.

Different transmission routes characterized by different transmission intensities are associated with both infection rates and other transmission‐related factors. All the factors that make mice dominant by enhancing the roles (or the relative roles) of vertical and direct transmissions would make evolution select for reduced virulence of *T. gondii* except increasing the predation rate and decreasing the decay rate of oocysts. The opposite effects of increasing the predation rate on vertical and direct transmission intensities make the evolutionary outcome depend on its net effect. Decreasing the decay rate of oocysts should have supported low virulent *T. gondii* similar to increasing its release rate, but actually depended on vertical transmission, which highlights the significance of evolutionary ecological feedback (Govaert et al., 2019; Lion, 2018; Lion & Metz, 2018). Under high vertical transmission, the dominance of mice cannot be achieved due to the disappearance of cat‐mouse interaction caused by the rapid decay of oocysts. This makes virulent *T. gondii* initially be selected to ensure that oocysts survive long enough until environmental infection occurs. Similarly, it should also be obtained that increasing the environmental infection rate of cats enhances the role of cats and that of oocysts thus favoring virulent *T. gondii*. However, this was not always the case, which can also be interpreted from this perspective. The loss of infected mice caused by virulent *T. gondii* cannot be compensated by the gain brought by low vertical transmission compared with high vertical transmission. In this case, a decrease in the mortality of infected mice induced by low virulent *T. gondii* is more efficient to maintain its quantity.

**FIGURE 8:**
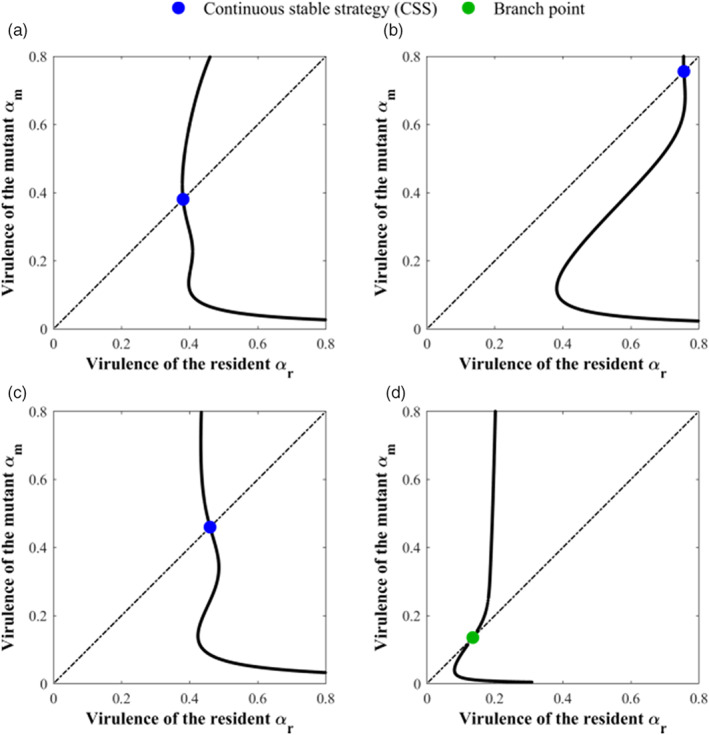
Qualitative verification of the present framework with the pairwise invisibility plots (PIPs) of *Toxoplasma gondii* virulence evolution in: (a) Europe, (b) Asia, (c) Africa, and (d) South America. Parameters in (a) u = 1.5, $\kappa_{\beta }$ = 0.5, $\rho_{2}$ = $\rho_{1}$ = 5 × 10^−4^, $\kappa_{q}$ = 1.5, $\kappa_{\theta }$ = 1.2, $\kappa_{\gamma_{c}}$ = $\kappa_{\gamma_{m}}$ = 1; (b) u = 0.5, $\rho_{2}$ = $\rho_{1}$ = 4 × 10^−4^, $\kappa_{\theta }$ = 1.5, and others are the same as those in (a); (c) u = 1, $\rho_{2}$ = $\rho_{1}$ = 6.5 × 10^−4^, $\kappa_{\theta }$ = 0.8, and others are the same as those in (a); (d) u = 2, $\rho_{2}$ = $\rho_{1}$ = 8 × 10^−4^, $\kappa_{\theta }$ = 0.4, $\kappa_{q}$ = 0.1, and others are the same as those in (a).

Our results indicate that the virulence evolution of *T. gondii* had a compromise between adapting to different transmission routes and maintaining the cat‐mouse interaction. This is similar to the combined effect of transmission route and host mortality on virulence evolution (Cressler et al., 2016; Williams & Day, 2001) and highlights the significance of evolutionary ecological feedback (Govaert et al., 2019; Lion, 2018; Lion & Metz, 2018). It was a response to the criterion that the direction of virulence evolution is to maximize its ability to infect more hosts (Geritz et al., 1998) without destroying the interaction in the complex cycle. Under this criterion, the different evolutionary scenarios can occur to balance the conflicting selection pressures. The evolutionary scenarios obtained herein can be used to describe the picture of *T. gondii* virulence in Europe, Asia, Africa, and South America, where the genotypes of *T. gondii* isolated from cats and mice are consistent (Amouei et al., 2020; Galeh et al., 2021; Table 3). A set of benchmark transmission‐related parameters representing a low virulence strain was chosen to investigate the virulence evolution of *T. gondii* in Europe (Shwab et al., 2014). There was only a dominant strain with low virulence in Figure 8a, which is just the situation in Europe. Compared with most countries in Europe, China, the main sampling place in Asia, has more pet cats (European Statistics, 2022; White Paper on China's Pet Industry, 2022), which limits cat activities but also provides a good living environment for them. This can be reflected by decreasing the predation rate and release rate and increasing the decay rate leading high virulent *T. gondii* to be dominant, which is in line with the fact that most strains of Chinese 1 are virulent (Chen et al., 2011; Figure 8b). There is an increase in the predation, release and decay rates due to more cats outdoors in a poor environment of Africa and the hot climate in the sampling areas from North Africa that is not conducive to oocyst survival (Hill & Dubey, 2002). In this situation, the dominance of low virulent *T. gondii* can also be obtained (Figure 8c). Given that South America has large sylvatic areas with a humid and warm climate that contributes to the long‐term survival of oocysts (Hill & Dubey, 2002), and that the vertical infection rate of atypical strains is up to 93% (Elsaid et al., 2001), a further increase in the predation, release, and vertical infection rates and a decrease in the decay rate was adopted in Figure 8d. The existence of the branch point implies the diversity of *T. gondii* virulence, which is just the case in South America. This qualitative verification of *T. gondii* virulence evolution in different areas by the present framework will provide a new perspective for the study of evolution.

**TABLE 3:** Dominant genotype and virulence of *Toxoplasma gondii* in different areas.

Area	Dominant genotype (Amouei et al., 2020; Galeh et al., 2021)	Virulence of dominant genotype (Chen et al., 2011; Shwab et al., 2016)
Europe	Type II	Avirulent
Asia	Chinese 1	Virulent
Africa	Type II, Type III	Avirulent
South America	No genotype appears to be clearly dominant	No dominant strain types

The results in Sections 3.1 and 3.2 suggest that evolutionary bifurcation easily occurred through cat‐mouse interaction caused by predation. The predation of cats on mice alters not only the number of mice as part of background mortality but also the composition of cats by preying on infected mice. This makes the evolutionary outcome more complicated than those studies that only regard it as part of host mortality and only consider the infection of prey (Choo et al., 2003; Morozov & Adamson, 2011; Morozov & Best, 2012). The general idea that increasing host mortality will select for increased virulence (Anderson & May, 1982) also holds if increased mortality is caused by the interaction with static predators in a classical SI model (Morozov & Adamson, 2011). However, when the predator is dynamic, the evolution of decreased virulence (Choo et al., 2003) and evolutionary bifurcation (Morozov & Best, 2012) are possible with a predation rate affected by infection. This highlights that there is a feedback loop between the degree of damage to the host by parasites and the background mortality of the host (Williams & Day, 2001). Here, such a feedback is reflected by the different impacts of predation on cats and mice resulting in the nonmonotonic change in the virulence of *T. gondii* with the predation rate and the occurrence of evolutionary bifurcation.

Furthermore, the global sensitivity analysis shows that changing the vertical infection rate of mice and the decay rate of oocysts was effective to regulate the virulence of *T. gondii*, decreasing the release rate of oocysts and increasing the vertical infection rate of mice made evolutionary diversity easy to occur. This demonstrates that controlling the characteristics of both mice and oocysts that act as the sources of infection is important and efficient for regulating the evolutionary outcome of *T. gondii*. When coinfection is further considered, the consistency of the evolutionary trend of *T. gondii* virulence reveals that the effect on the virulence of *T. gondii* of each factor is independent of coinfection. Nevertheless, the conclusion that the presence of coinfection favors virulent strain (Alizon & van Baalen, 2008) and complicates evolutionary ecological feedback making evolutionary bifurcation more likely to occur (Lion & Metz, 2018) can still be obtained.

Our study on the virulence evolution of *T. gondii* reveals that the effect of evolutionary ecological feedback involving multiple transmission routes and interactions between different hosts on evolutionary scenarios is far more complex than expected, and therefore deserves more attention.

## CONFLICT OF INTEREST

The authors declare no competing financial interests.

## Supporting information


Appendix S1

## Data Availability

The code and data supporting this article have been uploaded as part of the Supplementary Material.

## APPENDIX A The endemic equilibrium and the basic reproduction number of *Toxoplasma gondii*

The endemic equilibrium $W_{+}$ of the complex cycle can be written as

(A.1)
$$\begin{array}{c} {\hat{S}}_{c}=\frac{b_{c}E_{c}-\gamma_{c}\hat{P}}{d_{c}E_{c}};\;{\hat{I}}_{c}=\frac{\sigma \hat{P}}{\theta };\;{\hat{R}}_{c}=\frac{\xi {\hat{I}}_{c}}{d_{c}};\;{\hat{I}}_{m}=\frac{\gamma_{c}\hat{P}}{\rho_{2}\beta_{\mathrm{cm}}}\left(\frac{1}{{\hat{S}}_{c}E_{c}}-\frac{1}{1+\hat{P}}\right);\\ {\hat{S}}_{m}=\frac{b_{m}-\left(d_{m}+\alpha +\rho_{2}{\hat{N}}_{c}\right){\hat{I}}_{m}}{d_{m}+\rho_{1}{\hat{N}}_{c}};\;{\hat{N}}_{m}=\frac{b_{m}-\left(\alpha +\left(\rho_{2}-\rho_{1}\right){\hat{N}}_{c}\right){\hat{I}}_{m}}{d_{m}+\rho_{1}{\hat{N}}_{c}};\\ E_{c}=\frac{\gamma_{c}\theta }{\sigma \left(d_{c}+\xi \right)};D_{c}=\frac{\rho_{2}\beta_{\mathrm{cm}}}{d_{m}+\alpha +\rho_{2}{\hat{N}}_{c}};E_{m}=\frac{\gamma_{m}\theta }{\sigma \left(d_{c}+\xi \right)};V_{m}=\frac{b_{m}q_{m}}{d_{m}+\alpha +\rho_{2}{\hat{N}}_{c}} \end{array}$$

by setting the left side of Equation (1) to zero. Substituting $\;{\hat{I}}_{m}$ shown in Equation (A.1) into the fifth equation of Equation (1), one can obtain that

(A.2)
$$\left(1-\frac{{\hat{S}}_{c}E_{c}}{1+\hat{P}}\right)\left(1-\frac{V_{m}}{{\hat{N}}_{m}}\right)-\frac{{\hat{S}}_{c}D_{c}{\hat{S}}_{m}E_{m}}{1+\hat{P}}=0.$$

Combining Equation (A.1) and (A.2), the value of $\hat{P}$ can be solved and the endemic equilibrium $W_{+}$ can therefore be obtained.

According to van den Driessche and Watmough (van den Driessche & Watmough, 2002), the basic reproduction number $R_{0}$ is the spectral radius of the next‐generation matrix in the early stage of infection, $F{\left(V\right)}^{-1}$, which can be obtained by rewriting the second, fifth, and sixth equations of Equation (1) as

(A.3)

where $x=\left(x_{1},x_{2},x_{3}\right)=\left(I_{c},I_{m},P\right)$. In the early stage of infection, there is $\left(S_{c},I_{c},N_{c},S_{m},I_{m},N_{m},P\right)=\left(S_{c}^{0},0,N_{c}^{0},S_{m}^{0},0,N_{m}^{0},0\right)$. We compute

(A.4)
$$F={\left.\frac{\partial f\left(x\right)}{\partial x_{k}}\right|}_{k=1,2,3}=\left[\begin{array}{ccc} 0 & \rho_{2}\beta_{\mathrm{cm}}S_{c}^{0} & \gamma_{c}S_{c}^{0}\\ 0 & \frac{b_{m}q_{m}}{N_{m}^{0}} & \gamma_{m}S_{m}^{0}\\ 0 & 0 & 0 \end{array}\right]$$

and

(A.5)
$${\left(V\right)}^{-1}={\left({\left.\frac{\partial v\left(x\right)}{\partial x_{k}}\right|}_{k=1,2,3}\right)}^{-1}=\left[\begin{array}{ccc} \frac{1}{d_{c}+\xi } & 0 & 0\\ 0 & \frac{1}{d_{m}+\alpha +\rho_{2}N_{c}^{0}} & 0\\ \frac{\theta }{\sigma \left(d_{c}+\xi \right)} & 0 & \frac{1}{\sigma } \end{array}\right].$$

Therefore, the next‐generation matrix $F{\left(V\right)}^{-1}$ is

(A.6)
$$F{\left(V\right)}^{-1}=\left[\begin{array}{ccc} \frac{\gamma_{c}\theta S_{c}^{0}}{\sigma \left(d_{c}+\xi \right)} & \frac{\rho_{2}\beta_{\mathrm{cm}}S_{c}^{0}}{d_{m}+\alpha +\rho_{2}N_{c}^{0}} & \frac{\gamma_{c}S_{c}^{0}}{\sigma }\\ \frac{\gamma_{m}\theta S_{m}^{0}}{\sigma \left(d_{c}+\xi \right)} & \frac{b_{m}q_{m}}{N_{m}^{0}\left(d_{m}+\alpha +\rho_{2}N_{c}^{0}\right)} & \frac{\gamma_{m}S_{m}^{0}}{\sigma }\\ 0 & 0 & 0 \end{array}\right].$$

Then the basic reproduction number $R_{0}$ can be obtained,

(A.7)
$$\begin{array}{c} R_{0}=\rho \left[F{\left(V\right)}^{-1}\right]=\frac{1}{2}\left(S_{c}^{0}E_{c}+\frac{V_{m}}{N_{m}^{0}}\right)+\frac{1}{2}\sqrt{{\left(S_{c}^{0}E_{c}-\frac{V_{m}}{N_{m}^{0}}\right)}^{2}+4S_{c}^{0}D_{c}S_{m}^{0}E_{m}}\\ E_{c}=\frac{\gamma_{c}\theta }{\sigma \left(d_{c}+\xi \right)};D_{c}=\frac{\rho_{2}\beta_{\mathrm{cm}}}{d_{m}+\alpha +\rho_{2}N_{c}^{0}};E_{m}=\frac{\gamma_{m}\theta }{\sigma \left(d_{c}+\xi \right)};V_{m}=\frac{b_{m}q_{m}}{d_{m}+\alpha +\rho_{2}N_{c}^{0}} \end{array}$$

where $\rho_{R_{0}}\left[F{\left(V\right)}^{-1}\right]$ corresponds to the spectral radius of $F{\left(V\right)}^{-1}$.

## APPENDIX B The invasion reproduction number of *Toxoplasma gondii*

Similarly, the invasion reproduction number $R^{m}$ can also be derived by the next‐generation matrix method (van den Driessche & Watmough, 2002). At this time, the next‐generation matrix of the invasion system, $F^{m}{\left(V^{m}\right)}^{-1}$, can be obtained by rewriting Equation (4) as

(B.1)

where $x^{m}=\left(x_{1}^{m},x_{2}^{m},x_{3}^{m}\right)=\left(I_{c}^{\left(m\right)},I_{m}^{\left(m\right)},P^{\left(m\right)}\right)$. We compute the Jacobian matrix of $f^{m}\left(x^{m}\right)$ with respect to $x^{m}$

(B.2)
$$F^{m}={\left.\frac{\partial f^{m}\left(x^{m}\right)}{\partial x_{k}^{m}}\right|}_{k=1,2,3}=\left[\begin{array}{ccc} 0 & \rho_{2}\beta_{\mathrm{cm}}^{\left(m\right)}{\hat{S}}_{c} & \frac{\gamma_{c}^{\left(m\right)}{\hat{S}}_{c}}{1+\hat{P}}\\ 0 & \frac{b_{m}q_{m}^{\left(m\right)}}{{\hat{N}}_{m}} & \frac{\gamma_{m}^{\left(m\right)}{\hat{S}}_{m}}{1+\hat{P}}\\ 0 & 0 & 0 \end{array}\right]$$

and the inverse of the Jacobian matrix of $v^{m}\left(x^{m}\right)$ with respect to $x^{m}$

(B.3)
$${\left(V^{m}\right)}^{-1}={\left({\left.\frac{\partial v^{m}\left(x^{m}\right)}{\partial x_{k}^{m}}\right|}_{k=1,2,3}\right)}^{-1}=\left[\begin{array}{ccc} \frac{1}{d_{c}+\xi } & 0 & 0\\ 0 & \frac{1}{d_{m}+\alpha^{\left(m\right)}+\rho_{2}{\hat{N}}_{c}} & 0\\ \frac{\theta^{\left(m\right)}}{\sigma^{\left(m\right)}\left(d_{c}+\xi \right)} & 0 & \frac{1}{\sigma^{\left(m\right)}} \end{array}\right].$$

The next‐generation matrix of the invasion system $F^{m}{\left(V^{m}\right)}^{-1}$ is

(B.4)
$$F^{m}{\left(V^{m}\right)}^{-1}=\left[\begin{array}{ccc} \frac{\gamma_{c}^{\left(m\right)}\theta^{\left(m\right)}{\hat{S}}_{c}}{\sigma^{\left(m\right)}\left(d_{c}+\xi \right)\left(1+\hat{P}\right)} & \frac{\rho_{2}\beta_{\mathrm{cm}}^{\left(m\right)}{\hat{S}}_{c}}{d_{m}+\alpha^{\left(m\right)}+\rho_{2}{\hat{N}}_{c}} & \frac{\gamma_{c}^{\left(m\right)}{\hat{S}}_{c}}{\sigma^{\left(m\right)}\left(1+\hat{P}\right)}\\ \frac{\gamma_{m}^{\left(m\right)}\theta^{\left(m\right)}{\hat{S}}_{m}}{\sigma^{\left(m\right)}\left(d_{c}+\xi \right)\left(1+\hat{P}\right)} & \frac{b_{m}q_{m}^{\left(m\right)}}{{\hat{N}}_{m}\left(d_{m}+\alpha^{\left(m\right)}+\rho_{2}{\hat{N}}_{c}\right)} & \frac{\gamma_{m}^{\left(m\right)}{\hat{S}}_{m}}{\sigma^{\left(m\right)}\left(1+\hat{P}\right)}\\ 0 & 0 & 0 \end{array}\right].$$

Thus, the invasion reproduction number $R^{m}$ is

(B.5)
$$\begin{array}{c} R^{m}=\rho \left[F^{m}{\left(V^{m}\right)}^{-1}\right]=\frac{1}{2}\left(\frac{{\hat{S}}_{c}E_{c}^{\left(m\right)}}{1+\hat{P}}+\frac{V_{m}^{\left(m\right)}}{{\hat{N}}_{m}}\right)+\frac{1}{2}\sqrt{{\left(\frac{{\hat{S}}_{c}E_{c}^{\left(m\right)}}{1+\hat{P}}-\frac{V_{m}^{\left(m\right)}}{{\hat{N}}_{m}}\right)}^{2}+\frac{4{\hat{S}}_{c}D_{c}^{\left(m\right)}{\hat{S}}_{m}E_{m}^{\left(m\right)}}{1+\hat{P}}}\\ E_{c}^{\left(m\right)}=\frac{\gamma_{c}^{\left(m\right)}\theta^{\left(m\right)}}{\sigma^{\left(m\right)}\left(d_{c}+\xi \right)};D_{c}^{\left(m\right)}=\frac{\rho_{2}\beta_{\mathrm{cm}}^{\left(m\right)}}{d_{m}+\alpha^{\left(m\right)}+\rho_{2}{\hat{N}}_{c}};\\ E_{m}^{\left(m\right)}=\frac{\gamma_{m}^{\left(m\right)}\theta^{\left(m\right)}}{\sigma^{\left(m\right)}\left(d_{c}+\xi \right)};V_{m}^{\left(m\right)}=\frac{b_{m}q_{m}^{\left(m\right)}}{d_{m}+\alpha^{\left(m\right)}+\rho_{2}{\hat{N}}_{c}} \end{array}$$

where $\rho_{R^{m}}\left[F^{m}{\left(V^{m}\right)}^{-1}\right]$ corresponds to the spectral radius of $F^{m}{\left(V^{m}\right)}^{-1}$, $E_{c}^{\left(m\right)}$, $D_{c}^{\left(m\right)}$, $E_{m}^{\left(m\right)}$, and $V_{m}^{\left(m\right)}$ have similar implications as that mentioned in Section 2.1.

## APPENDIX C The invasion reproduction number of *Toxoplasma gondii* considering coinfection

In the presence of coinfection, given that $I_{c}^{\left(\mathrm{rm}\right)}\ll {\hat{I}}_{m}$, the second‐order terms related to $I_{c}^{\left(m\right)}I_{m}^{\left(\mathrm{rm}\right)}$ in Equation (10) can be omitted and the invasion system (Equation (10)) can be rewritten as

(C.1)
$$\begin{array}{l} \left(\begin{array}{c} \frac{{\mathrm{dI}}_{c}^{\left(m\right)}}{\mathrm{dt}}\\ \frac{{\mathrm{dI}}_{c}^{\left(\mathrm{rm}\right)}}{\mathrm{dt}}\\ \frac{{\mathrm{dI}}_{m}^{\left(m\right)}}{\mathrm{dt}}\\ \frac{{\mathrm{dI}}_{m}^{\left(\mathrm{rm}\right)}}{\mathrm{dt}}\\ \frac{{\mathrm{dP}}^{\left(m\right)}}{\mathrm{dt}} \end{array}\right)=\left(\begin{array}{c} \rho_{2}\beta_{\mathrm{cm}}^{\left(m\right)}{\hat{S}}_{c}\left(I_{m}^{\left(m\right)}+c_{m}I_{m}^{\left(\mathrm{rm}\right)}\right)+\frac{\gamma_{c}^{\left(m\right)}{\hat{S}}_{c}P^{\left(m\right)}}{1+\hat{P}}+c_{m}\xi I_{c}^{\left(\mathrm{rm}\right)}\\ h\left[\rho_{2}\beta_{\mathrm{cm}}I_{c}^{\left(m\right)}{\hat{I}}_{m}+\rho_{2}\beta_{\mathrm{cm}}^{\left(m\right)}{\hat{I}}_{c}\left(I_{m}^{\left(m\right)}+c_{m}I_{m}^{\left(\mathrm{rm}\right)}\right)+\frac{\gamma_{c}I_{c}^{\left(m\right)}\hat{P}+\gamma_{c}^{\left(m\right)}{\hat{I}}_{c}P^{\left(m\right)}}{1+\hat{P}}\right]\\ \frac{b_{m}q_{m}^{\left(m\right)}}{{\hat{N}}_{m}}\left(I_{m}^{\left(m\right)}+c_{m}I_{m}^{\left(\mathrm{rm}\right)}\right)+\frac{\gamma_{m}^{\left(m\right)}{\hat{S}}_{m}P^{\left(m\right)}}{1+\hat{P}}\\ h\left(\frac{\gamma_{m}I_{m}^{\left(m\right)}\hat{P}}{1+\hat{P}}+\frac{\gamma_{m}^{\left(m\right)}{\hat{I}}_{m}P^{\left(m\right)}}{1+\hat{P}}\right)\\ 0 \end{array}\right)\\ \;-\left(\begin{array}{c} \left(d_{c}+\xi \right)I_{c}^{\left(m\right)}+h\left[\rho_{2}\beta_{\mathrm{cm}}I_{c}^{\left(m\right)}{\hat{I}}_{m}+\frac{\gamma_{c}I_{c}^{\left(m\right)}\hat{P}}{1+\hat{P}}\right]\\ \left(d_{c}+\xi \right)I_{c}^{\left(\mathrm{rm}\right)}\\ \left(d_{m}+\alpha^{\left(m\right)}+\rho_{2}{\hat{N}}_{c}\right)I_{m}^{\left(m\right)}+\frac{h\gamma_{m}I_{m}^{\left(m\right)}\hat{P}}{1+\hat{P}}\\ \left(d_{m}+\alpha_{\mathrm{rm}}+\rho_{2}{\hat{N}}_{c}\right)I_{m}^{\left(\mathrm{rm}\right)}\\ \sigma^{\left(m\right)}P^{\left(m\right)}-\theta^{\left(m\right)}I_{c}^{\left(m\right)}-c_{m}\theta^{\left(m\right)}I_{c}^{\left(\mathrm{rm}\right)} \end{array}\right)\\ \;=f_{\mathrm{co}}^{m}\left(x_{\mathrm{co}}^{m}\right)-v_{\mathrm{co}}^{m}\left(x_{\mathrm{co}}^{m}\right)\; \end{array}$$

where $x_{\mathrm{co}}^{m}=\left(x_{\mathrm{co}1}^{m},x_{\mathrm{co}2}^{m},x_{\mathrm{co}3}^{m},x_{\mathrm{co}4}^{m},x_{\mathrm{co}5}^{m}\right)=\left(I_{c}^{\left(m\right)},I_{c}^{\left(\mathrm{rm}\right)},I_{m}^{\left(m\right)},I_{m}^{\left(\mathrm{rm}\right)},P^{\left(m\right)}\right)$. Therefore, the Jacobian matrix of $f_{\mathrm{co}}^{m}\left(x_{\mathrm{co}}^{m}\right)$ with respect to $x_{\mathrm{co}}^{m}$ is

(C.2)
$$F_{\mathrm{co}}^{m}={\left.\frac{\partial f_{\mathrm{co}}^{m}\left(x_{\mathrm{co}}^{m}\right)}{\partial x_{\mathrm{co}k}^{m}}\right|}_{k=1,\ldots ,5}=\left[\begin{array}{ccccc} 0 & c_{m}\xi & \rho_{2}\beta_{\mathrm{cm}}^{\left(m\right)}{\hat{S}}_{c} & c_{m}\rho_{2}\beta_{\mathrm{cm}}^{\left(m\right)}{\hat{S}}_{c} & \frac{\gamma_{c}^{\left(m\right)}{\hat{S}}_{c}}{1+\hat{P}}\\ M_{c} & 0 & h\rho_{2}\beta_{\mathrm{cm}}^{\left(m\right)}{\hat{I}}_{c} & c_{m}h\rho_{2}\beta_{\mathrm{cm}}^{\left(m\right)}{\hat{I}}_{c} & \frac{h\gamma_{c}^{\left(m\right)}{\hat{I}}_{c}}{1+\hat{P}}\\ 0 & 0 & \frac{b_{m}q_{m}^{\left(m\right)}}{{\hat{N}}_{m}} & \frac{c_{m}b_{m}q_{m}^{\left(m\right)}}{{\hat{N}}_{m}} & \frac{\gamma_{m}^{\left(m\right)}{\hat{S}}_{m}}{1+\hat{P}}\\ 0 & 0 & M_{m} & 0 & \frac{h\gamma_{m}^{\left(m\right)}{\hat{I}}_{m}}{1+\hat{P}}\\ 0 & 0 & 0 & 0 & 0 \end{array}\right]..$$

In Equation (C.2), $M_{c}=h\rho_{2}\beta_{\mathrm{cm}}{\hat{I}}_{m}+\frac{h\gamma_{c}\hat{P}}{1+\hat{P}}$ and $M_{m}=\frac{h\gamma_{m}\hat{P}}{1+\hat{P}}$. The inverse of the Jacobian matrix of $v_{\mathrm{co}}^{m}\left(x_{\mathrm{co}}^{m}\right)\;$ with respect to $x_{\mathrm{co}}^{m}$ is

(C.3)
$${\left(V_{\mathrm{co}}^{m}\right)}^{-1}={\left({\left.\frac{\partial v_{\mathrm{co}}^{m}\left(x_{\mathrm{co}}^{m}\right)\;}{\partial x_{\mathrm{co}k}^{m}}\right|}_{k=1,\ldots ,5}\right)}^{-1}=\left[\begin{array}{ccccc} \frac{1}{\psi_{c_{1}}} & 0 & 0 & 0 & 0\\ 0 & \frac{1}{\psi_{c_{2}}} & 0 & 0 & 0\\ 0 & 0 & \frac{1}{\psi_{m_{1}}} & 0 & 0\\ 0 & 0 & 0 & \frac{1}{\psi_{m_{2}}} & 0\\ \frac{\theta^{\left(m\right)}}{\sigma^{\left(m\right)}\psi_{c1}} & \frac{c_{m}\theta^{\left(m\right)}}{\sigma^{\left(m\right)}\psi_{c_{2}}} & 0 & 0 & \frac{1}{\sigma^{\left(m\right)}} \end{array}\right]$$

where

(C.4)
$$\begin{array}{c} \psi_{c_{1}}=d_{c}+\xi +M_{c};\psi_{c_{2}}=d_{c}+\xi ;\\ \psi_{m_{1}}=d_{m}+\alpha^{\left(m\right)}+\rho_{2}{\hat{N}}_{c}+M_{m};\psi_{m_{2}}=d_{m}+\alpha_{\mathrm{rm}}+\rho_{2}{\hat{N}}_{c} \end{array}.$$

Therefore, $F_{\mathrm{co}}^{m}{\left(V_{\mathrm{co}}^{m}\right)}^{-1}$, the next‐generation matrix of the invasion system considering coinfection, is shown as follows.

(C.5)
$$F_{\mathrm{co}}^{m}{\left(V_{\mathrm{co}}^{m}\right)}^{-1}=\left[\begin{array}{ccccc} \frac{\gamma_{c}^{\left(m\right)}{\hat{S}}_{c}\theta^{\left(m\right)}}{\sigma^{\left(m\right)}\psi_{c_{1}}\left(1+\hat{P}\right)} & \frac{c_{m}\gamma_{c}^{\left(m\right)}{\hat{S}}_{c}\theta^{\left(m\right)}}{\sigma^{\left(m\right)}\psi_{c_{2}}\left(1+\hat{P}\right)}+\frac{c_{m}\xi }{\psi_{c_{2}}} & \frac{\rho_{2}\beta_{\mathrm{cm}}^{\left(m\right)}{\hat{S}}_{c}}{\psi_{m_{1}}} & \frac{c_{m}\rho_{2}\beta_{\mathrm{cm}}^{\left(m\right)}{\hat{S}}_{c}}{\psi_{m_{2}}} & \frac{\gamma_{c}^{\left(m\right)}{\hat{S}}_{c}}{\sigma^{\left(m\right)}\left(1+\hat{P}\right)}\\ \frac{h\gamma_{c}^{\left(m\right)}{\hat{I}}_{c}\theta^{\left(m\right)}}{\sigma^{\left(m\right)}\psi_{c_{1}}\left(1+\hat{P}\right)}+\frac{M_{c}}{\psi_{c_{1}}} & \frac{c_{m}h\gamma_{c}^{\left(m\right)}{\hat{I}}_{c}\theta^{\left(m\right)}}{\sigma^{\left(m\right)}\psi_{c_{2}}\left(1+\hat{P}\right)} & \frac{h\rho_{2}\beta_{\mathrm{cm}}^{\left(m\right)}{\hat{I}}_{c}}{\psi_{m_{1}}} & \frac{c_{m}h\rho_{2}\beta_{\mathrm{cm}}^{\left(m\right)}{\hat{I}}_{c}}{\psi_{m_{2}}} & \frac{h\gamma_{c}^{\left(m\right)}{\hat{I}}_{c}}{\sigma^{\left(m\right)}\left(1+\hat{P}\right)}\\ \frac{\gamma_{m}^{\left(m\right)}{\hat{S}}_{m}\theta^{\left(m\right)}}{\sigma^{\left(m\right)}\psi_{c_{1}}\left(1+\hat{P}\right)} & \frac{c_{m}\gamma_{m}^{\left(m\right)}{\hat{S}}_{m}\theta^{\left(m\right)}}{\sigma^{\left(m\right)}\psi_{c_{2}}\left(1+\hat{P}\right)} & \frac{b_{m}q_{m}^{\left(m\right)}}{{\hat{N}}_{m}\psi_{m_{1}}} & \frac{c_{m}b_{m}q_{m}^{\left(m\right)}}{{\hat{N}}_{m}\psi_{m_{2}}} & \frac{\gamma_{m}^{\left(m\right)}{\hat{S}}_{m}}{\sigma^{\left(m\right)}\left(1+\hat{P}\right)}\\ \frac{h\gamma_{m}^{\left(m\right)}{\hat{I}}_{m}\theta^{\left(m\right)}}{\sigma^{\left(m\right)}\psi_{c_{1}}\left(1+\hat{P}\right)} & \frac{c_{m}h\gamma_{m}^{\left(m\right)}{\hat{I}}_{m}\theta^{\left(m\right)}}{\sigma^{\left(m\right)}\psi_{c_{2}}\left(1+\hat{P}\right)} & \frac{M_{m}}{\psi_{m_{1}}} & 0 & \frac{h\gamma_{m}^{\left(m\right)}{\hat{I}}_{m}}{\sigma^{\left(m\right)}\left(1+\hat{P}\right)}\\ 0 & 0 & 0 & 0 & 0 \end{array}\right]$$

The invasion reproduction number in this case is equal to the spectral radius of $F_{\mathrm{co}}^{m}{\left(V_{\mathrm{co}}^{m}\right)}^{-1}$, that is, $R_{\mathrm{co}}^{m}=\rho_{R_{\mathrm{co}}^{m}}\left[F_{\mathrm{co}}^{m}{\left(V_{\mathrm{co}}^{m}\right)}^{-1}\right]$, which can be solved numerically.

## References

[eva13530-bib-0001] Ajai, V. , Seon‐Kyeong, K. , Giacomini, N. , Boothroyd, J. C. , & Sapolsky, R. M. (2007). Behavioral changes induced by toxoplasma infection of rodents are highly specific to aversion of cat odors. Proceedings of the National Academy of Sciences of the United States of America, 104(15), 6442–6447. 17404235 10.1073/pnas.0608310104 PMC1851063

[eva13530-bib-0002] Alizon, S. , & van Baalen, M. (2008). Multiple infections, immune dynamics, and the evolution of virulence. The American Naturalist, 172(4), 150–168. 10.1086/590958 18702601

[eva13530-bib-0003] Amouei, A. , Sarvi, S. , Sharif, M. , Aghayan, S. A. , Javidnia, J. , Mizani, A. , Moosazadeh, M. , Shams, N. , Hosseini, S. A. , Hosseininejad, Z. , Nayeri Chegeni, T. , Badali, H. , & Daryani, A. (2020). A systematic review of *Toxoplasma gondii* genotypes and feline: Geographical distribution trends. Transboundary and Emerging Diseases, 67(1), 46–64. 31464067 10.1111/tbed.13340

[eva13530-bib-0004] Anderson, R. M. , & May, R. M. (1982). Coevolution of hosts and parasites. Parasitology, 85(2), 411–426. 6755367 10.1017/s0031182000055360

[eva13530-bib-0005] Auld, S. , Searle, C. L. , & Duffy, M. A. (2017). Parasite transmission in a natural multihost‐multiparasite community. Philosophical Transactions of the Royal Society B: Biological Sciences, 372(1719), 20160097. 10.1098/rstb.2016.0097 PMC5352823 28289264

[eva13530-bib-0006] Baker, P. J. , Ansell, R. J. , Dodds, P. A. A. , Webber, C. E. , & Harris, S. (2003). Factors affecting the distribution of small mammals in an urban area. Mammal Review, 33(1), 95–100.

[eva13530-bib-0007] Baker, P. J. , Bentley, A. J. , Ansell, R. J. , & Harris, S. (2005). Impact of predation by domestic cats *Felis catus* in an urban area. Mammal Review, 35(3–4), 302–312.

[eva13530-bib-0008] Barratt, D. G. (1998). Predation by house cats, Felis catus (L.), in Canberra, Australia. II. Factors affecting the amount of prey caught and estimates of the impact on wildlife. Wildlife Research, 25(5), 475–487.

[eva13530-bib-0009] Betancourt, M. , Escriu, F. , Fraile, A. , & García‐Arenal, F. (2013). Virulence evolution of a generalist plant virus in a heterogeneous host system. Evolutionary Applications, 6(6), 875–890. 24062798 10.1111/eva.12073 PMC3779090

[eva13530-bib-0010] Boldin, B. , & Kisdi, E. (2012). On the evolutionary dynamics of pathogens with direct and environmental transmission. Evolution, 66(8), 2514–2527. 22834749 10.1111/j.1558-5646.2012.01613.x

[eva13530-bib-0011] Bonhoeffer, S. , Lenski, R. E. , & Ebert, D. (1996). The curse of the pharaoh: The evolution of virulence in pathogens with long living propagules. Proceedings of the Royal Society B, 263(1371), 715–721. 8763793 10.1098/rspb.1996.0107

[eva13530-bib-0012] Brown, S. P. , Cornforth, D. M. , & Mideo, N. (2012). Evolution of virulence in opportunistic pathogens: Generalism, plasticity, and control. Trends in Microbiology, 20(7), 336–342. 22564248 10.1016/j.tim.2012.04.005 PMC3491314

[eva13530-bib-0013] Caraco, T. , & Wang, I. N. (2008). Free‐living pathogens: Life‐history constraints and strain competition. Journal of Theoretical Biology, 250(3), 569–579. 18062992 10.1016/j.jtbi.2007.10.029 PMC2262931

[eva13530-bib-0014] Cen, X. , Feng, Z. , & Zhao, Y. (2014). Emerging disease dynamics in a model coupling within‐host and between‐host systems. Journal of Theoretical Biology, 361, 141–151. 25093825 10.1016/j.jtbi.2014.07.030

[eva13530-bib-0015] Chen, Z. W. , Gao, J. M. , Huo, X. X. , Wang, L. , Yu, L. , Halm‐Lai, F. , Xu, Y. H. , Song, W. J. , Hide, G. , Shen, J. L. , & Lun, Z. R. (2011). Genotyping of *Toxoplasma gondii* isolates from cats in different geographic regions of China. Veterinary Parasitology, 183(1–2), 166–170. 21757292 10.1016/j.vetpar.2011.06.013

[eva13530-bib-0016] Choo, K. , Williams, P. D. , & Day, T. (2003). Host mortality, predation and the evolution of parasite virulence. Ecology Letters, 6(4), 310–315.

[eva13530-bib-0017] Courchamp, F. , Pontier, D. , Langlais, M. , & Artois, M. (1995). Population dynamics of feline immunodeficiency virus within cat populations. Journal of Theoretical Biology, 175(4), 553–560. 7475091 10.1006/jtbi.1995.0163

[eva13530-bib-0018] Cressler, C. E. , McLeod, D. V. , Rozins, C. , van den Hoogen, J. , & Day, T. (2016). The adaptive evolution of virulence: A review of theoretical predictions and empirical tests. Parasitology, 143(7), 915–930. 26302775 10.1017/S003118201500092X PMC4873896

[eva13530-bib-0019] Day, T. (2002). Virulence evolution via host exploitation and toxin production in spore‐producing pathogens. Ecology Letters, 5(4), 471–476.

[eva13530-bib-0020] Deng, H. , Cummins, R. , Schares, G. , Trevisan, C. , Enemark, H. , Waap, H. , Srbljanovic, J. , Djurkovic‐Djakovic, O. , Pires, S. M. , van der Giessen, J. W. B. , & Opsteegh, M. (2021). Mathematical modelling of *Toxoplasma gondii* transmission: A systematic review. Food and Waterborne Parasitology, 22, 22. 10.1016/j.fawpar.2020.e00102 PMC7753131 33364472

[eva13530-bib-0021] Dercole, F. , & Rinaldi, S. (2008). Analysis of evolutionary processes: The adaptive dynamics approach and its applications (pp. 76–85). Princeton University Press.

[eva13530-bib-0022] Dubey, J. P. (1996). Infectivity and pathogenicity of *Toxoplasma gondii* oocysts for cats. The Journal of Parasitology, 82(6), 957–961. 8973406

[eva13530-bib-0023] Dubey, J. P. (1998). Advances in the life cycle of *Toxoplasma gondii* . International Journal for Parasitology, 28(7), 1019–1024. 9724872 10.1016/s0020-7519(98)00023-x

[eva13530-bib-0024] Dubey, J. P. (2001). Oocyst shedding by cats fed isolated bradyzoites and comparison of infectivity of bradyzoites of the VEG strain *Toxoplasma gondii* to cats and mice. The Journal of Parasitology, 87(1), 215–219. 11227895 10.1645/0022-3395(2001)087[0215:OSBCFI]2.0.CO;2

[eva13530-bib-0025] Dubey, J. P. (2006). Comparative infectivity of oocysts and bradyzoites of *Toxoplasma gondii* for intermediate (mice) and definitive (cats) hosts. Veterinary Parasitology, 140(1–2), 69–75. 16647212 10.1016/j.vetpar.2006.03.018

[eva13530-bib-0026] Dubey, J. P. (2010). Toxoplasmosis of animals and humans. CRC Press.

[eva13530-bib-0027] Dubey, J. P. , & Frenkel, J. K. (1972). Cyst‐induced toxoplasmosis in cats. The Journal of Protozoology, 19(1), 155–177. 5008846 10.1111/j.1550-7408.1972.tb03431.x

[eva13530-bib-0028] Dubey, J. P. , & Frenkel, J. K. (1973). Experimental toxoplasma iInfection in mice with strains producing oocysts. The Journal of Parasitology, 59(3), 505–512. 4576142

[eva13530-bib-0029] Dubey, J. P. , & Frenkel, J. K. (1976). Feline toxoplasmosis from acutely infected mice and the development of toxoplasma cysts. The Journal of Protozoology, 23(4), 537–546. 1003342 10.1111/j.1550-7408.1976.tb03836.x

[eva13530-bib-0030] Dubey, J. P. , & Frenkel, J. K. (1998). Toxoplasmosis of rats: A review, with considerations of their value as an animal model and their possible role in epidemiology. Veterinary Parasitology, 77(1), 1–32. 9652380 10.1016/s0304-4017(97)00227-6

[eva13530-bib-0031] Dubey, J. P. , & Hoover, E. A. (1977). Attempted transmission of *Toxoplasma gondii* infection from pregnant cats to their kittens. Journal of the American Veterinary Medical Association, 170(5), 538–540. 557468

[eva13530-bib-0032] Dubey, J. P. , Lindsay, D. S. , & Speer, C. A. (1998). Structures of *Toxoplasma gondii* tachyzoites, bradyzoites, and sporozoites and biology and development of tissue cysts. Clinical Microbiology Reviews, 11(2), 267–299. 9564564 10.1128/cmr.11.2.267 PMC106833

[eva13530-bib-0033] Dubey, J. P. , Speer, C. A. , Shen, S. K. , Kwok, O. C. H. , & Blixt, J. A. (1997). Oocyst‐induced murine toxoplasmosis: Life cycle, pathogenicity, and stage conversion in mice fed *Toxoplasma gondii* oocysts. The Journal of Parasitology, 83(5), 870–882. 9379292

[eva13530-bib-0034] Elsaid, M. M. A. , Martins, M. S. , Frézard, F. , Braga, E. M. , & Vitor, R. W. A. (2001). Vertical toxoplasmosis in a murine model, protection after immunization with antigens of *Toxoplasma gondii* incorporated into liposomes. Memórias do Instituto Oswaldo Cruz, 96(1), 99–104. 11285480 10.1590/s0074-02762001000100011

[eva13530-bib-0035] European Statistics . (2022). https://www.fediaf.org/who‐we‐are/european‐statistics.html

[eva13530-bib-0036] Feng, Z. , Velasco‐Hernandez, J. , & Tapia‐Santos, B. (2013). A mathematical model for coupling within‐host and between‐host dynamics in an environmentally‐driven infectious disease. Mathematical Biosciences, 241(1), 49–55. 23041478 10.1016/j.mbs.2012.09.004

[eva13530-bib-0037] Frank, S. A. (1996). Models of parasite virulence. The Quarterly Review of Biology, 71(1), 37–78. 8919665 10.1086/419267

[eva13530-bib-0038] Galal, L. , Sarr, A. , Cuny, T. , Brouat, C. , Coulibaly, F. , Sembène, M. , Diagne, M. , Diallo, M. , Sow, A. , Hamidović, A. , Plault, N. , Dardé, M. L. , Ajzenberg, D. , & Mercier, A. (2019). The introduction of new hosts with human trade shapes the extant distribution of *Toxoplasma gondii* lineages. Plos Neglected Tropical Diseases, 13(7), e0007435. 31295245 10.1371/journal.pntd.0007435 PMC6622481

[eva13530-bib-0039] Galeh, T. M. , Sarvi, S. , Hosseini, S. A. , & Daryani, A. (2021). Genetic diversity of *Toxoplasma gondii* isolates from rodents in the world: A systematic review. Transboundary and Emerging Diseases, 69(3), 943–957. 33825346 10.1111/tbed.14096

[eva13530-bib-0040] Gandon, S. (2004). Evolution of multihost parasites. Evolution, 58(3), 455–469. 15119430

[eva13530-bib-0041] Geritz, S. A. H. , Kisdi, E. , Meszéna, G. , & Metz, J. A. J. (1998). Evolutionarily singular strategies and the adaptive growth and branching of the evolutionary tree. Evolutionary Ecology, 12(1), 35–57.

[eva13530-bib-0042] González‐Parra, G. C. , Arenas, A. J. , Aranda, D. F. , Villanueva, R. J. , & Jódar, L. (2009). Dynamics of a model of toxoplasmosis disease in human and cat populations. Computers & Mathematcs with Applications, 57(10), 1692–1700.

[eva13530-bib-0043] Gotteland, C. , McFerrin, B. M. , Zhao, X. , Gilot‐Fromont, E. , & Lélu, M. (2014). Agricultural landscape and spatial distribution of *Toxoplasma gondii* in rural environment: An agent‐based model. International Journal of Health Geographics, 13(1), 45. 25352091 10.1186/1476-072X-13-45 PMC4271439

[eva13530-bib-0044] Govaert, L. , Fronhofer, E. A. , Lion, S. , Eizaguirre, C. , Bonte, D. , Egas, M. , Hendry, A. P. , de Brito Martins, A. , Melián, C. J. , Raeymaekers, J. A. M. , Ratikainen, I. I. , Saether, B. E. , Schweitzer, J. A. , & Matthews, B. (2019). Eco‐evolutionary feedbacks‐theoretical models and perspectives. Functional Ecology, 33(1), 13–30.

[eva13530-bib-0045] Guidot, A. , Jiang, W. , Ferdy, J. B. , Thébaud, C. , Barberis, P. , Gouzy, J. , & Genin, S. (2014). Multihost experimental evolution of the pathogen Ralstonia solanacearum unveils genes involved in adaptation to plants. Molecular Biology and Evolution, 31(11), 2913–2928. 25086002 10.1093/molbev/msu229

[eva13530-bib-0046] Hide, G. (2016). Role of vertical transmission of *Toxoplasma gondii* in prevalence of infection. Expert Review of Anti‐Infective Therapy, 14(3), 335–344. 26807498 10.1586/14787210.2016.1146131

[eva13530-bib-0047] Hill, D. , & Dubey, J. P. (2002). *Toxoplasma gondii*: Transmission, diagnosis and prevention. Clinical Microbiology and Infection, 8(10), 634–640. 12390281 10.1046/j.1469-0691.2002.00485.x

[eva13530-bib-0048] Hill, D. E. , Chirukandoth, S. , & Dubey, J. P. (2005). Biology and epidemiology of *Toxoplasma gondii* in man and animals. Animal Health Research Reviews, 6(1), 41–61. 16164008 10.1079/ahr2005100

[eva13530-bib-0049] Jiang, W. , Sullivan, A. M. , Su, C. , & Zhao, X. (2012). An agent‐based model for the transmission dynamics of *Toxoplasma gondii* . Journal of Theoretical Biology, 293, 15–26. 22004993 10.1016/j.jtbi.2011.10.006

[eva13530-bib-0050] Kamiya, T. , Mideo, N. , & Alizon, S. (2018). Coevolution of virulence and immunosuppression in multiple infections. Journal of Evolutionary Biology, 31(7), 995–1005. 29668109 10.1111/jeb.13280

[eva13530-bib-0051] Kawecki, T. J. (1994). Accumulation of deleterious mutations and the evolutionary cost of being a generalist. The American Naturalist, 144(5), 833–838.

[eva13530-bib-0052] Kays, R. W. , & DeWan, A. A. (2004). Ecological impact of inside/outside house cats around a suburban nature preserve. Animal Conservation, 7, 273–283.

[eva13530-bib-0053] Koutou, O. , Traore, B. , & Sangaré, B. (2021). Analysis of schistosomiasis global dynamics with general incidence functions and two delays. International Journal of Applied and Computational Mathematics, 7(6), 245.

[eva13530-bib-0054] Lelu, M. , Langlais, M. , Poulle, M. L. , & Gilot‐Fromont, E. (2010). Transmission dynamics of *Toxoplasma gondii* along an urban‐rural gradient. Theoretical Population Biology, 78(2), 139–147. 20685358 10.1016/j.tpb.2010.05.005

[eva13530-bib-0055] Lievens, E. J. P. , Perreau, J. , Agnew, P. , Michalakis, Y. , & Lenormand, T. (2018). Decomposing parasite fitness reveals the basis of specialization in a two‐host, two‐parasite system. Evolution Letters, 2(4), 390–405. 30283690 10.1002/evl3.65 PMC6121826

[eva13530-bib-0056] Lion, S. (2018). Theoretical approaches in evolutionary ecology: Environmental feedback as a unifying perspective. The American Naturalist, 191(1), 21–44. 10.1086/694865 29244555

[eva13530-bib-0057] Lion, S. , & Metz, J. A. J. (2018). Beyond R‐0 maximisation: On pathogen evolution and environmental dimensions. Trends in Ecology & Evolution, 33(6), 458–473. 29665966 10.1016/j.tree.2018.02.004

[eva13530-bib-0058] Lipsitch, M. , Siller, S. , & Nowak, M. A. (1996). The evolution of virulence in pathogens with vertical and horizontal transmission. Evolution, 50(5), 1729–1741. 28565576 10.1111/j.1558-5646.1996.tb03560.x

[eva13530-bib-0059] Morozov, A. , & Best, A. (2012). Predation on infected host promotes evolutionary branching of virulence and pathogens' biodiversity. Journal of Theoretical Biology, 307, 29–36. 22579552 10.1016/j.jtbi.2012.04.023

[eva13530-bib-0060] Morozov, A. Y. , & Adamson, M. W. (2011). Evolution of virulence driven by predator‐prey interaction: Possible consequences for population dynamics. Journal of Theoretical Biology, 276(1), 181–191. 21320512 10.1016/j.jtbi.2011.02.007

[eva13530-bib-0061] Mosquera, J. , & Adler, F. R. (1998). Evolution of virulence: A unified framework for coinfection and superinfection. Journal of Theoretical Biology, 195(3), 293–313. 9826485 10.1006/jtbi.1998.0793

[eva13530-bib-0062] Pei, Y. , Ji, X. , Li, C. , & Gao, S. (2018). Dynamics of a model of toxoplasmosis disease in cat and human with varying size populations. Mathematics and Computers in Simulation, 144, 52–59.

[eva13530-bib-0063] Regoes, R. R. , Nowak, M. A. , & Bonhoeffer, S. (2000). Evolution of virulence in a heterogeneous host population. Evolution, 54(1), 64–71. 10937184 10.1111/j.0014-3820.2000.tb00008.x

[eva13530-bib-0064] Rigby, M. C. , & Jokela, J. (2000). Predator avoidance and immune defence: Costs and trade‐offs in snails. Philosophical Transactions of the Royal Society B: Biological Sciences, 267(1439), 171–176. 10.1098/rspb.2000.0983 PMC1690515 10687823

[eva13530-bib-0065] Savadogo, A. , Sangare, B. , & Ouedraogo, H. (2022). A mathematical analysis of prey‐predator population dynamics in the presence of an SIS infectious disease. Research in Mathematics, 9(1), 2020399.

[eva13530-bib-0066] Shwab, E. K. , Jiang, T. , Pena, H. F. J. , Gennari, S. M. , Dubey, J. P. , & Su, C. (2016). The ROP18 and ROP5 gene allele types are highly predictive of virulence in mice across globally distributed strains of *Toxoplasma gondii* . International Journal for Parasitology, 46(2), 141–146. 26699401 10.1016/j.ijpara.2015.10.005

[eva13530-bib-0067] Shwab, E. K. , Saraf, P. , Zhu, X. Q. , Zhou, D. H. , McFerrin, B. M. , Ajzenberg, D. , Schares, G. , Hammond‐Aryee, K. , van Helden, P. , Higgins, S. A. , Gerhold, R. W. , Rosenthal, B. M. , Zhao, X. , Dubey, J. P. , & Su, C. (2018). Human impact on the diversity and virulence of the ubiquitous zoonotic parasite *Toxoplasma gondii* . Proceedings of the National Academy of Sciences of the United States of America, 115(29), E6956–E6963. 29967142 10.1073/pnas.1722202115 PMC6055184

[eva13530-bib-0068] Shwab, E. K. , Zhu, X.‐Q. , Majumdar, D. , Pena, H. F. , Gennari, S. M. , Dubey, J. P. , & Su, C. (2014). Geographical patterns of *Toxoplasma gondii* genetic diversity revealed by multilocus PCR‐RFLP genotyping. Parasitology, 141(4), 453–461. 24477076 10.1017/S0031182013001844

[eva13530-bib-0069] Taylor, L. H. , Latham, S. M. , & Woolhouse, M. E. J. (2001). Risk factors for human disease emergence. Philosophical Transactions of the Royal Society B, 356(1411), 983–989. 10.1098/rstb.2001.0888 PMC1088493 11516376

[eva13530-bib-0070] Tenter, A. M. , Heckeroth, A. R. , & Weiss, L. M. (2000). *Toxoplasma gondii*: From animals to humans. International Journal for Parasitology, 30(12–13), 1217–1258. 11113252 10.1016/s0020-7519(00)00124-7 PMC3109627

[eva13530-bib-0071] Traore, B. , Koutou, O. , & Sangaré, B. (2019). Global dynamics of a seasonal mathematical model of schistosomiasis transmission with general incidence function. Journal of Biological Systems, 27(1), 19–49.

[eva13530-bib-0072] Turner, M. , Lenhart, S. , Rosenthal, B. , & Zhao, X. (2013). Modeling effective transmission pathways and control of the world's most successful parasite. Theoretical Population Biology, 86(5), 50–61. 23624067 10.1016/j.tpb.2013.04.001

[eva13530-bib-0073] van den Driessche, P. , & Watmough, J. (2002). Reproduction numbers and sub‐threshold endemic equilibria for compartmental models of disease transmission. Mathematical Biosciences, 180, 29–48. 12387915 10.1016/s0025-5564(02)00108-6

[eva13530-bib-0074] White Paper on China's Pet Industry . (2022). https://www.sohu.com/a/518200180_99982343

[eva13530-bib-0075] Williams, P. D. , & Day, T. (2001). Interactions between sources of mortality and the evolution of parasite virulence. Philosophical Transactions of the Royal Society B: Biological Sciences, 268(1483), 2331–2337. 10.1098/rspb.2001.1795 PMC1088884 11703873

[eva13530-bib-0076] Woolhouse, M. E. J. , Taylor, L. H. , & Haydon, D. T. (2001). Population biology of multihost pathogens. Science, 292(5519), 1109–1112. 11352066 10.1126/science.1059026

